# Investigation of the Dietary Preferences of Two Dorid Nudibranchs by Feeding-Choice Experiments and Chemical Analysis

**DOI:** 10.1007/s10886-023-01444-z

**Published:** 2023-07-17

**Authors:** Lauren Gris, Christopher N. Battershill, Michele R. Prinsep

**Affiliations:** 1 Chemistry and Applied Physics, School of Science, University of Waikato, Private Bag 3105, 3240 Hamilton, New Zealand; 2 University of Waikato Coastal Marine Field Station, 58 Cross Road, Sulphur Point, 3110 Tauranga, New Zealand

**Keywords:** Nudibranch, *Goniobranchus aureomarginatus*, *Ceratosoma amoenum*, Sponges, Feeding-choice experiments, Dictyodendrin

## Abstract

Feeding-choice experiments were conducted under laboratory conditions with two dorid spongivorous nudibranchs, *Goniobranchus aureomarginatus* and *Ceratosoma amoenum*, collected from a sponge meadow off Tauranga, New Zealand with two sponge prey (*Dysidea teawanui* sp.nov. and an undescribed species from the Dictyodendrillidae family, possibly *Dictyodendrilla tenella* (Lendenfeld 1888). The first choice of prey, the total number of prey choices made, and the time spent on each prey target was recorded, results indicating that each nudibranch had strong preferences for specific prey species. Preferences were significant when the time spent grazing on prey was taken into consideration. *Goniobranchus aureomarginatus* had a strong preference for the undescribed Dictyodendrillid sponge, while *Ceratosoma ameonum* preferred *Dysidea teawanui*. The results of the feeding-choice experiments matched observations in the wild. Chemical analysis of the undescribed Dictyodendrillid sponge led to the isolation and characterisation of six known bioactive metabolites, dictyodendrin C (**1**), D (**2**) and F (**3**), as well as denigrin E (**4**), dactylpyrrole A (**5**) and lamellarin O1 (**6**). Two of the known compounds, dictyodendrins C (**1**) and F (**3**) were also isolated from *G. aureomarginatus* individuals. Chemical analysis of *D. teawanui* afforded ergosterol peroxide, 5α,8α-epidioxy-24-methylcholesta-6,22-dien-3β-ol (**7**). The structures of the isolated natural products were elucidated based on extensive analysis of 1D and 2D NMR data.

**Supplementary Information:**

The online version contains supplementary material available at 10.1007/s10886-023-01444-z.

## Introduction

Dorid nudibranchs (Order: Nudibranchia, Family: Dorididae) are slow-moving, shell-less, marine gastropod mollusks that feed on sessile prey, among which some species exclusively feed on sponges (Cimino et al. 1999; López-Acosta et al. 2023). Some sponge species are chemically defended and represent an important source of biologically active secondary metabolites, containing unique structures and displaying great diversity (Hong et al. 2022). Some dorid nudibranchs have developed resistance strategies to feed upon chemically defended sponges and can sequester secondary metabolites via selective feeding and accumulation for their own defence (Chen et al. 2023). This ability is believed to be associated with the need to acquire a chemical defence due to shell loss (Faulkner et al. 1983). The study of specific predator-prey relationships in dorid nudibranchs is especially attractive from a chemistry perspective for the discovery of new bioactive compounds, as well as from an ecological point of view, to provide a better understanding of chemical defences, feeding behaviour and to support environment monitoring and management (Chen et al. 2023; Dean et al. 2017). While both specialist and generalist feeders have been identified amongst nudibranchs (Picton et al. 1996), feeding-choice experiments can be used to determine preferential feeding behaviour, particularly in coral-eating nudibranchs (Hall et al. 1982; Hoover et al. 2012; Ritson-Williams et al. 2003). To date, the diet of dorid nudibranchs has been examined primarily via field studies (Chu et al. 2012; Gemballa et al. 2004; Megina et al. 2002). However, few feeding-choice experiments have been conducted under laboratory conditions on spongivorous dorid nudibranchs. To our knowledge, only the dietary preferences of the Antarctic nudibranch *Doris kerguelenensis* (Iken et al. 2002), and *Glossodoris pallida* from Guam have been assessed (Rogers et al. 1991).

To extend knowledge of predator-prey relationships for spongivorous nudibranchs, two dorid nudibranchs, *Goniobranchus aureomarginatus* and *Ceratosoma amoenum*, were studied. Both species are known to feed on sponges (Willan et al. 2020) and were collected from a sponge meadow within Tauranga Harbour, New Zealand. The sponge meadow supports several species of sponge, among which is the recently reported species *Dysidea teawanui* (Order: Dictyoceratida, Family: Dysideidae) (Mc Cormack et al. 2020), and an as yet undescribed black sponge from the Dictyodendrillidae Family (Order: Dendroceratida), that has some diagnostic skeletal similarities to *Dictyodendrilla tenella* (Bergquist 1996). From field observations, *C. amoenum*, a common nudibranch found in Australasian waters, is known to predate on *D. teawanui* (Mc Cormack et al. 2020) and collected specimens were found to be grazing on the distinctive, large blue sponge. The second nudibranch species studied, *G. aureomarginatus*, is endemic to New Zealand (Willan et al. 2020) and half of the specimens collected were found grazing on the Dictyodendrillid sponge whilst the other half were found on the seabed but not grazing on any specific substrate, presumably transiting to find more prey.

To precisely assess any dietary preferences of both nudibranch species towards the two sponge species, feeding-choice experiments were conducted under laboratory conditions with freshly collected sponge and nudibranch specimens. Separate samples of the *Dysidea* and the Dictyodendrillid sponges were then extracted with a mixture of methanol and dichloromethane. The crude extracts were then separated by reversed phase chromatography, followed by either gel filtration or normal phase chromatography to isolate the secondary metabolites present. The structures of the isolated natural products were elucidated based on extensive analysis of 1D and 2D NMR data. In this paper, we wish to report the results of the feeding-choice experiments, as well as the preliminary chemical analyses.

## Methods and Materials

### General Experimental Procedures

Lyophilisation of the crude extracts utilized a BÜCHI lyophilizer Lyovapor L-200. Solvent was removed under reduced pressure using a BÜCHI Rotavapor 011 Rotary Evaporator Condenser system combined with a Thermo Haake K10 circulating chiller. Solvents used for general purposes were purchased as drum grade and distilled in the laboratory. Water used for chemical analyses was Type 1 grade. The reversed phase purifications were carried out with C18 YMC-gel ODS-A (YMC). The gel filtrations were carried out on Sephadex LH-20. The normal phase purification was carried out on silica (Davisil, 60 Å, 35–70 μ). Specific rotation was recorded on an Optical Activity AA-5 polarimeter. NMR experiments were performed on a JEOL ECZR 600 MHz spectrometer and are referenced to either CD_3_OD (^1^ H : 3.31 ppm, ^13^ C : 49.0 ppm) or CDCl_3_ (^1^ H : 7.26ppm, ^13^ C : 77.16 ppm). Delta NMR Data Processing Software (v5.3.3) was used for spectral analyses. ESI mass spectra were recorded using a Bruker Daltonics MicrOTOF electrospray ionisation mass spectrometer. Sodium formate solution was used for calibration. Solid samples were dissolved in a few drops drop of MeOH, and one drop of each sample was added to an Eppendorf tube pre-filled with 1.5 mL of MeOH. Samples were centrifuged before use to ensure separation of undissolved solids. Spectra were recorded with a Capillary Exit voltage between 90 and 150 V in positive ion mode and between − 90 V and − 150 V in negative ion mode.

### Species Studied 

#### *Goniobranchus aureomarginatus*

*Goniobranchus aureomarginatus* (Cheeseman, 1881) is endemic to New Zealand and displays a brilliant orange-yellow band on the edge of the mantle. Adult specimens usually reach a length of 3 to 5 cm and commonly live on rocky reefs, between 5 and 10 m deep (Willan et al. 2020). *Ceratosoma amoenum* (Cheeseman, 1886), also known as the clown nudibranch, can be found in Australian and New Zealand waters. Large and vivid orange spots covering a predominantly white mantle make it easily recognisable, even though the size and number of spots varies considerably between individuals. *C. amoenum* specimens can be found on rocky reefs at a depth of 5 to 15 m. Adult specimens can reach 6 cm long, although 2 to 3 cm is more common. Both species are known to feed on sponge and were identified from the e-guide on New Zealand marine biota “Super sea slugs, a guide to the sea slugs of New Zealand” developed by the National Institute of Water and Atmospheric Research (NIWA) marine taxonomy group (Willan et al. 2020). A voucher specimen of each studied nudibranch is kept at the University of Waikato in Hamilton, New Zealand.

### Collection and Maintenance of the Samples

All samples were collected at depths between 2 and 6 m using free diving and scuba diving between March and December 2022 at Dive Crescent, a sponge meadow within Tauranga Harbour, New Zealand. A total of six *G. aureomarginatus* specimens were collected; three of them were found grazing on the Dictyodendrillid sponge and three roaming across rock and broken shell substrate. The majority of *G. aureomarginatus* specimens collected had black stomach contents which were clearly visible at the time of collection. A total of four *C. amoenum* specimens were collected in October 2022 with the majority either found on *D. teawanui* or on the Dictyodendrillid sponge. During a separate collection trip at the same location, pieces of each studied sponge and seven *G. aureomarginatus* specimens were also collected and directly frozen. A voucher specimen of each studied sponge is kept at the University of Waikato Coastal Marine Field Station in Tauranga, New Zealand. Nudibranchs and sponge specimens used for feeding-choice experiments were kept alive in ambient seawater. The seawater was oxygenated with a portable air pump at the Aquatic research centre at Waikato University, Hamilton, NZ. Nudibranch species were kept separately, with individual specimens of each species either kept separately or in pairs, for 1 to 2 days prior to experiments and starved. Experiments were conducted in a room kept at 20 °C (close to ambient sea temperature at the time).

### Feeding-Choice Experiments

**Fig. 1:**
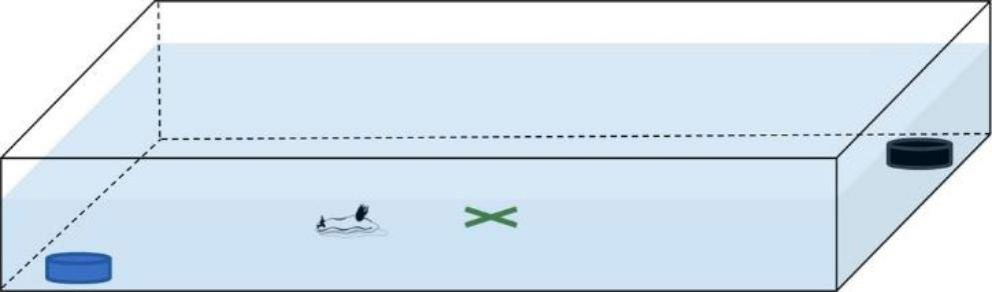
Feeding-choice experiment set-up with both prey placed at opposite corners. The green cross represents the nudibranch starting point

Feeding-choice experiments were conducted under laboratory conditions. Experiments with *G. aureomarginatus* were carried out on six individuals (n = 6), with between eight and eleven experiments conducted, for a total experiment duration of 94 to 124 h for each individual. Eleven experiments were conducted on four *C. amoenum* individuals (n = 4) and lasted for a total of 109 h. The tanks used were either a Perspex tank (45 cm x 23 cm x 4.5 cm) or plastic containers (35 cm x 20 cm x 10 cm). Artificial seawater was prepared using Crystal Sea®Marinemix to a salinity between 35 and 36 ppt. The depth of water was set a few cm lower on the Perspex tank to allow comparable volumes of water across the tanks and was such that the organisms were completely submerged in all instances. Prior to each experiment, the nudibranchs were given an acclimation period of 5 to 10 min and placed at the centre of the tank. Either one or two *G. aureomarginatus* individuals were placed per tank and each individual remained in the same tank for each experiment. The four *C. amoenum* individuals were placed in the same tank. Before the start of the experiment, the nudibranchs were carefully placed back in the centre of the tank and each piece of sponge was placed at opposite corners of the tank (Fig. 1). For the Perspex tank, sponge pieces were placed a few cm away from the corners to allow comparable distance between the prey across the tanks. For each sponge species, the main sponge specimen was kept under oxygenated seawater in a separate container. Portions of sponge of similar size (2 to 3 cm in diameter) were used. Sponge pieces used for the feeding-choice experiments were taken from the main sponge specimen and exposed to the air for the shortest amount of time possible while being transferred to the tanks for the experiments. No sign of deterioration in their condition were observed during the experiments. Water was oxygenated during the experiments. Experiments were carried out in natural light during the day and during the night, artificial light was provided by a LED Batten fixed above the tanks. Each experiment was recorded using the timelapse mode of a GoPro Hero 8 camera. A choice for a sponge was counted when the mouth of the nudibranch was in contact with the prey, as determined by extensive analysis of the recorded videos. After each experiment, the tanks were emptied and rinsed thoroughly with tap water, before being filled again with artificial seawater. Fresh pieces of prey were used at the beginning of each experiment and the placement of prey was alternated between experiments. No specimens died during the experiments.

### Chemical Analysis

#### Dictyodendrillid sponge

A portion of the frozen Dictyodendrillid sponge (80 g) was soaked overnight in MeOH/DCM (3:1), then blended, and filtered under vacuum. The extraction steps were repeated several times with a 1 h soaking time until the filtrate was colourless. The filtrates were combined, and the solvent was removed under reduced pressure. The crude extract was then lyophilised to afford 3.1 g of black powder, which was purified by bench column chromatography on reversed phase C18 with a steep, stepped gradient from H_2_O to MeOH to DCM. The fractions that eluted with H_2_O/MeOH (1:1) (179.3 mg) and H_2_O/MeOH (3:7) (77.2 mg) were further purified separately on Sephadex LH-20 with 100% MeOH. Several fractions from each column were then further separately purified on Sephadex LH-20 with H_2_O/MeOH (1:1) to afford the known compounds dictyodendrin C (**1**, 9.5 mg) and D (**2**, 6.1 mg). The fraction that eluted with H_2_O/MeOH (1:9) from the initial C18 chromatography column (36.1 mg) was further purified on Sephadex LH-20 with 100% MeOH to afford denigrin E (**4**, 5 mg).

Several minor compounds were also detected and to afford their isolation and characterisation, a larger scale extraction of the Dictyodendrillid sponge (417 g) was carried out following identical steps as described above and 13.2 g of crude extract was obtained. The extract was divided into three equal parts and each part was purified separately by bench column chromatography on reversed phase C18 with a steep, stepped gradient from H_2_O to MeOH to DCM. Three fractions that eluted with H_2_O/MeOH (3:7) or H_2_O/MeOH (1:9) (278.3 mg) were then recombined and further purified on reversed phase C18 with a similar gradient. The fractions that eluted with 100% MeOH to 100% DCM (135.7 mg) were recombined and purified further on Sephadex LH-20 with 100% MeOH to afford dictyodendrin F (**3**, 7.5 mg). Three fractions that eluted with H_2_O/MeOH (3:7) on the initial C18 column were recombined (178.7 mg) and further purified on reversed phase C18 with a similar gradient. The fraction that eluted with H_2_O/MeOH (3:7) (74.1 mg) was further purified on Sephadex LH-20 with 100% MeOH to afford lamellarin O1 (**6**, 3.2 mg). Several fractions that eluted with H_2_O/MeOH (1:1) on the initial reversed phase C18 chromatography column were purified separately on Sephadex LH-20 with H_2_O/MeOH (1:1) and similar late eluting fractions from that column were then recombined and purified further on Sephadex LH-20 with H_2_O/MeOH (3:7) to afford dactylpyrrole A (**5**, 0.2 mg).

#### *Goniobranchus aureomarginatus*

The seven whole *G. aureomarginatus* individuals, previously collected at the same location and directly frozen (8.8 g), were jointly extracted following the steps described above. The crude extract was dried under a nitrogen flow to afford 602.8 mg of dry extract, which was purified by bench column chromatography on reversed phase C18 with a steep, stepped gradient from H_2_O to MeOH to DCM. The fraction that eluted with H_2_O/MeOH (1:9) (7.1 mg) was further purified separately on Sephadex LH-20 with H_2_O/MeOH (7:3) to afford dictyodendrin C (**1**, 0.5 mg) and F (**3**, 0.5 mg).

#### *Dysidea teawanui*

A portion of the frozen *D. teawanui* sponge (260 g) was extracted following the steps described above. The crude extract was then lyophilised to afford 9.25 g of dry powder, which was divided into two parts; each part was separately purified by bench column chromatography on reversed phase C18 with a steep, stepped gradient from H_2_O to MeOH to DCM. One fraction that eluted with 100% MeOH (56.6 mg) was further purified on normal phase chromatography (Davisil, 60 Å, 35–70 μ) to afford ergosterol peroxide, 5α,8α-epidioxy-24-methylcholesta-6,22-dien-3β-ol (**7**, 3.1 mg).

##### Dictyodendrin C (1)

brown-yellow, amorphous solid; ESI(+)MS *m/z* 681 [M + Na]^+^, ESI(-)MS *m/z* 635 [M-Na]^−^ (Figures S1a and S1b); ^1^ H and ^13^ C NMR data (CD_3_OD) are a close match to the literature data for dictyodendrin C (Table S1) (Warabi et al. 2003).

##### Dictyodendrin D (2)

brown-yellow, amorphous solid; ESI(+)MS *m/z* 783 [M + Na]^+^, ESI(-)MS *m/z* 737 [M-Na]^−^, 357 [M/2-Na]^−^ (Figures S2a and S2b); ^1^ H and ^13^ C NMR data (CD_3_OD) are a close match to the literature data for dictyodendrin D (Table S2) (Warabi et al. 2003).

##### Dictyodendrin F (3)

green-brown, amorphous solid; ESI(+)MS *m/z* 579 [M + Na]^+^, 1135 [2 M + Na]^+^, ESI(-)MS *m/z* 555 [M-H]^−^, 1111 [2 M-H]^−^ (Figures S3a and S3b); ^1^ H NMR data (CD_3_OD) are a close match to the literature data for dictyodendrin F (Table S3) (Zhang et al. 2012a, b).

##### Denigrin E (4)

magenta, amorphous solid; ESI(+)MS *m/z* 584 [M + H]^+^, 606 [M + Na]^+^, 1189 [2 M + Na]^+^; ESI(-)MS *m/z* 582 [M-H]^−^, 1165 [2 M-H]^−^ (Figures S4a and S4b); ^1^ H NMR data (CD_3_OD) are a close match to the literature data for denigrin E (Table S4) (Kang et al. 2020).

##### Dactylpyrrole A (5)

yellow, amorphous solid; ESI(+)MS *m/z* 530 [M + Na]^+^, 1037 [2 M + Na]^+^, ESI(-)MS *m/z* 506 [M-H]^−^, 1013 [2 M-H]^−^ (Figures S5a and S5b), ^1^ H NMR data (CD_3_OD) are a close match to the literature data for dactylpyrrole A (Table S5) (Kang et al. 2020).

##### Lamellarin O1 (6)

ESI(+)MS *m/z* 466 [M + Na]^+^, 909 [2 M + Na]^+^, ESI(-)MS *m/z* 442 [M-H]^−^, 885 [2 M-H]^−^ (Figures S6a and S6b), ^1^ H and ^13^ C NMR data (CD_3_OD) are a close match to the literature data for lamellarin O1 (Table S6) (Zhang et al. 2012a, b).

##### 5α,8α-epidioxy-24-methylcholesta-6,22-diene-3β-ol (7)

[α]^20^_D_ -30.4° (CHC1_3_); ESI(+)MS *m/z* 429 [M + H]^+^ (Figure S7); ^1^ H and ^13^ C NMR data (CDCl_3_) are a close match to the literature data for 5α,8α-epidioxy-24-methylcholesta-6,22-diene-3β-ol (Table S7).

## Results

### Feeding-Choice Experiments

Due to nudibranchs being slow-moving animals, experiments of several hours and with no flow were conducted. In previous feeding-choice studies on spongivorous nudibranchs conducted under laboratory conditions (Iken et al. 2002; Rogers et al. 1991), only the frequency of choices towards a specific sponge species was recorded, either with a flow through or no flow apparatus. However, such an analysis, does not consider whether the nudibranchs stayed on the sponge they initially chose or whether they changed their position. In our case, feeding choice in both nudibranch species was analysed in three different ways. Firstly, only the initial choice the nudibranchs made between the offered sponge species was considered and the observed choice frequencies were compared to those expected from a random distribution (*2-tailed binomial* test, α = 0.05, P = 0.5). Secondly, to allow for whether individuals stayed on the sponge they initially chose or subsequently changed their position after the first choice, the total number of times a sponge species was chosen was considered, and the observed frequencies were compared to expected frequencies from a random distribution using a *Chi-squared* test (α = 0.05). Finally, the total time each specimen spent grazing on each prey was considered and the observed frequencies were compared to expected frequencies from a random distribution using a *Chi-squared* test (*Chi-squared* test, α = 0.05).

### *Goniobranchus aureomarginatus*

*G. aureomarginatus* specimens (n = 6) were offered a choice between the Dictyodendrillid sponge and *D. teawanui*. If the first choice the nudibranchs made between the offered sponge species is considered, a total of 35 choices were recorded (Table 1). Neither sponge was significantly preferred (Dictyodendrillid sponge: 20 choices, *D. teawanui* : 15 choices; P = 0.499). Each nudibranch specimen made at least two first choices throughout the duration of the experiments. The results were similar when the total number of times a sponge species was chosen were accounted for. A total of 62 choices were recorded (Table 1) and no nudibranchs showed significant preference for one of the sponges (Dictyodendrillid sponge: 37 choices, *D. teawanui* : 25 choices, χ^2^ = 2.323).

**Table 1:** *G. aureomarginatus*^*1*^ feeding-choice experiments with two sponge species as offered prey. Number of choices considering the first choice^2^ and the total number of choices.^3^

*G. aureomarginatus*	Number of choices				
	Dictyodendrillid sponge	*D. teawanui*	*Degree of freedom (df)*	χ^2^	Significance
First choice	20	15			P = 0.4990
All choices counted	37	25	1	2.32	P = 0.1275

^1^n = 6.

^2^Number of choices considering the first choice (2-tailed binomial test, α = 0.05, P = 0.5).

^3^Total number of choices (Chi-squared test, α = 0.05).

**Fig. 2:**
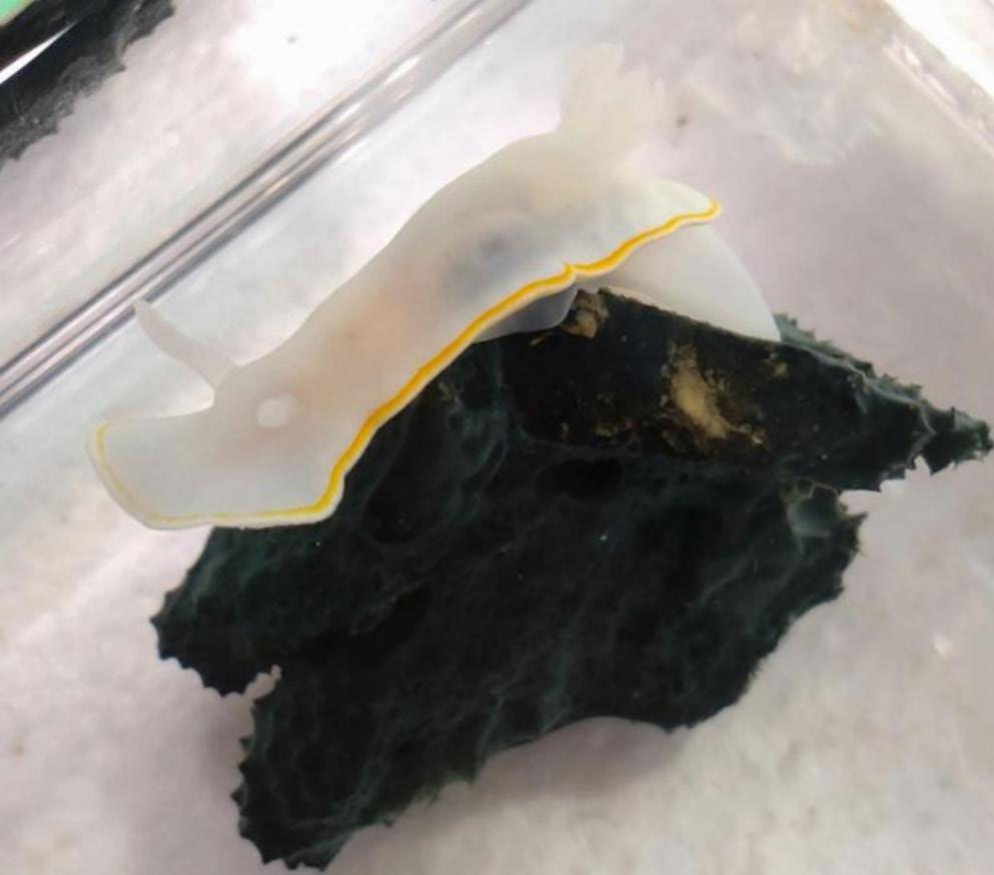
*G. aureomarginatu*s crawling on the Dictyodendrillid sponge during the feeding-choice experiments

**Fig. 3:**
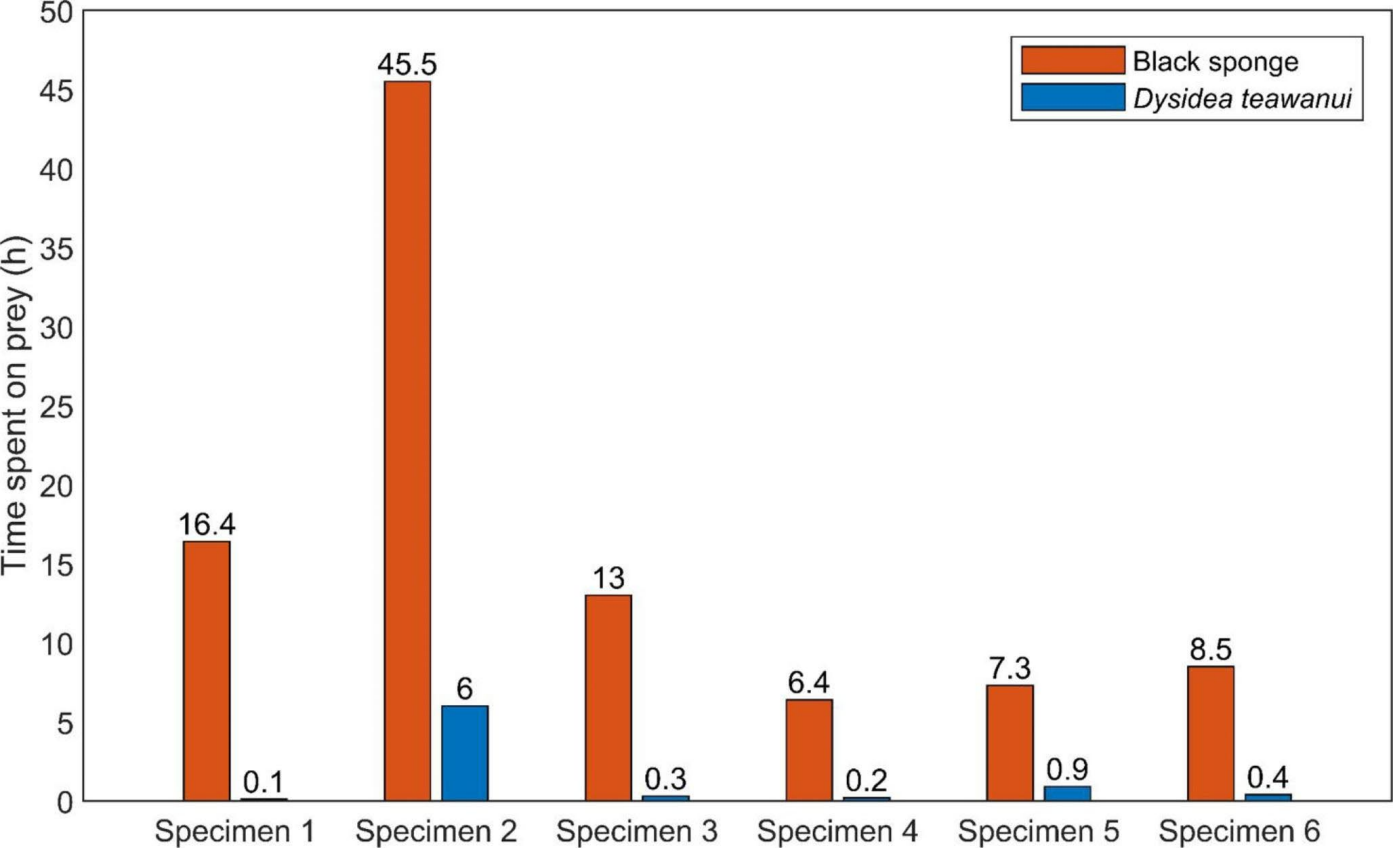
*G. aureomarginatus* (n = 6). Time spent on prey for each specimen across the feeding-choice experiments

When the total time each specimen spent grazing on each prey was considered (Table 2), the results were significantly different, with the Dictyodendrillid sponge being significantly preferred. The difference between the observed and expected frequencies was extremely statistically significant (χ^2^ = 73.96 for the cumulative time spent on prey for all the specimens, n = 6). The total time spent on prey was 105 h and during the remaining hours, the nudibranchs crawled, either at the bottom of the tank or on the walls. Out of the 105 h spent on both prey, 97.2 h were spent on the Dictyodendrillid sponge (93%) (Figs. 2) and 7.9 h on *D. teawanui* (7%). All *G. aureomarginatus* specimens spent between 88 and 99% of their time grazing on the Dictyodendrillid sponge. Individual nudibranchs could be recognised by their size and/or shape, and the behaviour of each individual nudibranch was analysed (Fig. 3). The strong preference for the Dictyodendrillid sponge was consistent in every individual (57.76 < χ^2^ < 96.04). Across the whole duration of the experiment, each specimen spent between 7 and 41% of their time on prey. The time spent on each prey for each *G. aureomarginatus* specimen per individual experiment can be found in Table S8.

**Table 2:** *G. aureomarginatu*s^1^ feeding-choice experiment with two sponge species as offered prey. Frequency of the total time on spent on prey considered.^2^

*G. aureomarginatus* specimens	Dictyodendrillid sponge		*D. teawanui*					
	Time spent on prey (h)	Frequency (%)	Time spent on prey (h)	Frequency (%)	Cumulative time spent on both prey (h)	*df*	χ^2^	Significance
1	16.4	99	0.1	1	16.5	1	96.04	P < 0.0001
2	45.5	88	6.0	12	51.5	1	57.76	P < 0.0001
3	13.0	98	0.3	2	13.3	1	92.16	P < 0.0001
4	6.4	98	0.2	2	6.6	1	92.16	P < 0.0001
5	7.3	89	0.9	11	8.2	1	60.84	P < 0.0001
6	8.5	96	0.4	4	8.9	1	84.64	P < 0.0001
Cumulative time spent on prey (h)	97.2	93	7.9	7	105.0	1	73.96	P < 0.0001

^1^n = 6

^2^Chi-squared test, α = 0.05

### *Ceratosoma amoenum*

*C. amoenum* specimens (n = 4) were offered a choice between Dictyodendrillid sponge and *D. teawanui*. A total of 16 first choices were recorded. Neither sponge was significantly preferred (Dictyodendrillid sponge: 9 choices, *D. teawanui*: 7 choices; P = 0.8036). The results were similar when the total number of times a sponge species was chosen were accounted for. A total of 19 choices were recorded (Table 3) but no nudibranch showed a significant preference for one of the sponges (Dictyodendrillid sponge: 11 choices, *D. teawanui*: 8 choices, χ^2^ = 0.474). Each nudibranch specimen made at least 3 choices throughout the duration of the experiments.

**Table 3:** *C. amoenum*^*1*^ feeding-choice experiments with two sponge species as offered prey. Number of choices considering the first choice^2^ and the total number of choices.^3^

* C. amoenum*	Number of choices				
	Dictyodendrillid sponge	*D. teawanui*	*Degree of freedom (df)*	χ^2^	Significance
First choice	9	7			P = 0.8036
All choices counted	11	8	1	0.47	P = 0.4913

^1^n = 6

^2^2-tailed binomial test, α = 0.05, P = 0.5

^3^Chi-squared test, α = 0.05

**Fig. 4:**
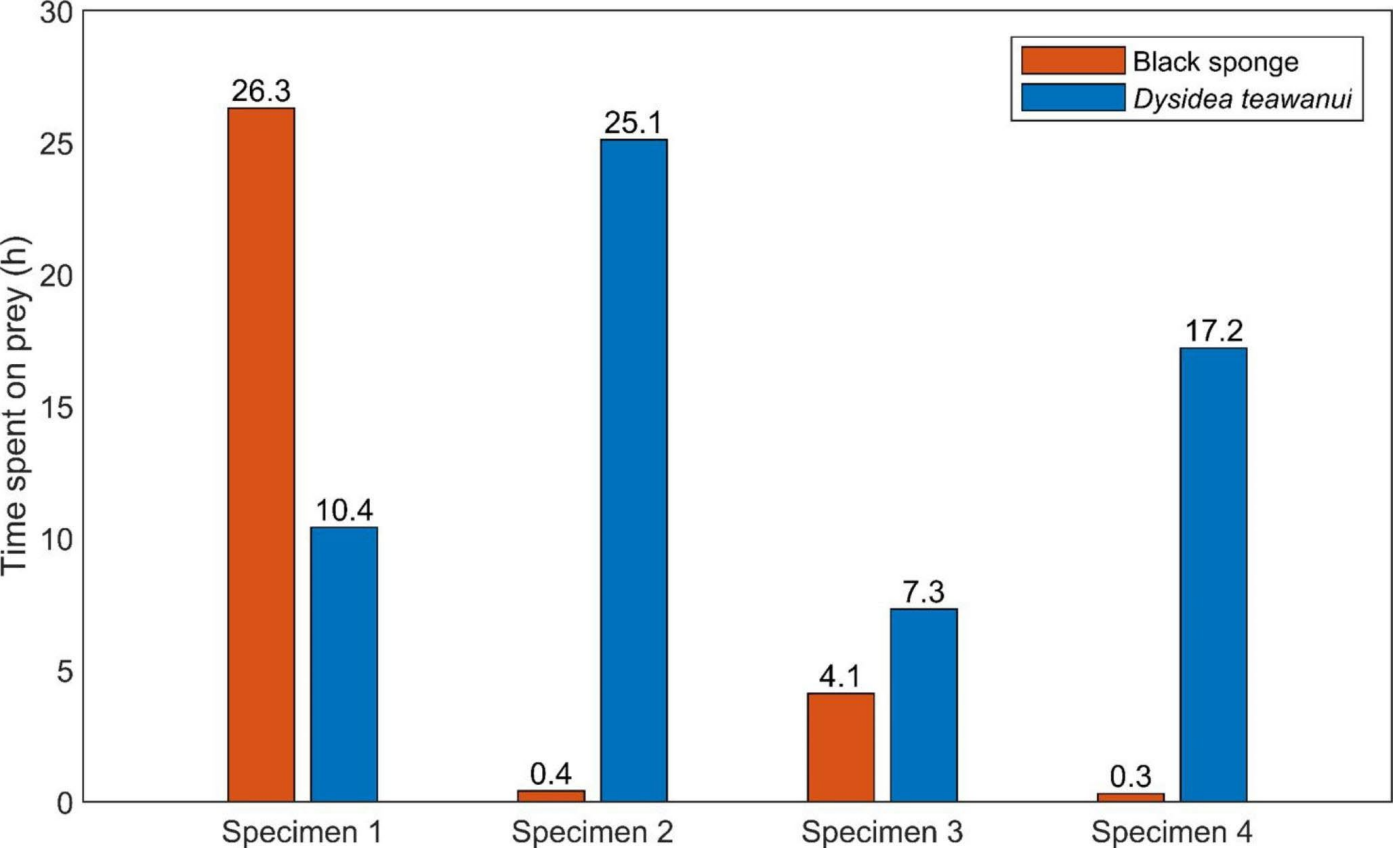
*C. amoenum* (n = 4). Time spent on prey for each specimen across the feeding-choice experiments

**Fig. 5:**
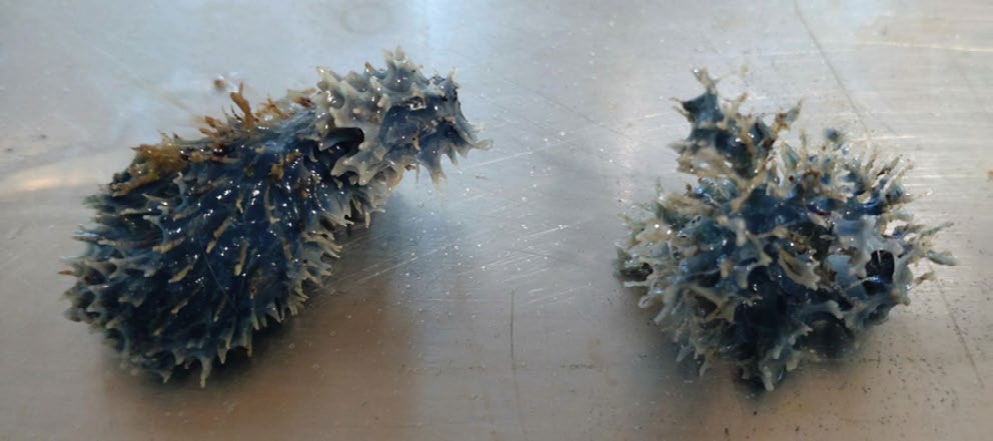
*D. teawanui* fragments before (left) and after (right) a feeding-choice experiment with *C. amoenum*

When the total time each specimen spent grazing on each prey was considered (Table 4), the results indicated that *C. amoenum* significantly prefers *D. teawanui*, with the difference between the observed and expected frequency being statistically significant (χ^2^ = 10.240 for the cumulative time spent on prey for all the specimens, n = 4). The total time spent on prey was 91.1 h and during the remaining hours, the nudibranchs crawled either at the bottom of the tank or on the walls. Out of the 91.1 h spent on both prey, 31.1 h were spent on the Dictyodendrillid sponge (34%) and 60 h on *D. teawanui* (66%). The behaviour of each individual nudibranch was analysed (Fig. 4), and this preference was consistent in every individual (7.8 < χ^2^ < 92.16), except one that significantly preferred the Dictyodendrillid sponge (χ^2^ = 19.36). Across the whole duration of the experiment, each specimen spent between 10 and 34% of their time on prey. The scars on *D. teawanui*, caused by the nudibranchs’ grazing, confirmed that the nudibranchs were feeding on the prey (Fig. 5). The time spent on each prey for each *C. amoenum* specimen per individual experiment can be found in Table S9.

**Table 4:** *C. amoenum*^1^ feeding-choice experiment with two sponge species as offered prey. Frequency of the total time on spent on prey considered.^2^

* C. amoenum* specimens	Dictyodendrillid sponge		*D. teawanui*					
	Time spent on prey (h)	Frequency (%)	Time spent on prey (h)	Frequency (%)	Total time spent on prey (h)	*df*	χ^2^	Significance
1	26.3	72	10.4	28	36.7	1	19.36	P < 0.0001
2	0.4	2	25.1	98	25.5	1	92.16	P < 0.0001
3	4.1	36	7.3	64	11.4	1	7.84	P = 0.0051
4	0.3	2	17.2	98	17.5	1	92.16	P < 0.0001
Cumulative time spent on prey (h)	31.1	34	60.0	66	91.1	1	10.24	P = 0.0014

^1^n = 6

^2^Chi-squared test, α = 0.05

### Chemical Analysis

**Fig. 6:**
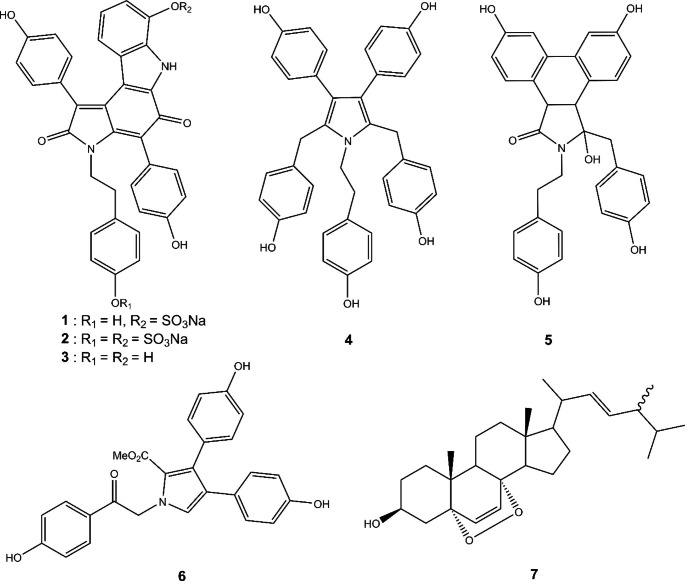
Structures of the isolated natural products

The extremely strong preference of *G. aureomarginatus* for the Dictyodendrillid sponge piqued our interest in this specific sponge, hence chemical analysis of the sponge was conducted. A piece of the sponge was extracted with a mixture of methanol and dichloromethane. The crude extract was then separated by reversed phase chromatography, followed by repeated size exclusion chromatography to afford the known compounds dictyodendrin C (**1**, 9.5 mg) and D (**2**, 6.1 mg), as well as denigrin E (**4**, 5 mg) (Fig. 6). Several minor compounds were detected via thin layer chromatography (TLC) and Liquid Chromatography-Mass Spectrometry (LCMS) but could not be isolated due to their small quantity. A larger scale extraction of the Dictyodendrillid sponge was conducted following similar extraction and purification steps as the first extraction. This resulted in the isolation and characterisation of an additional three known compounds: dictyodendrin F (**3**, 7.5 mg), dactylpyrrole A (**5**, 0.2 mg) and lamellarin O1 (**6**, 3.2 mg) (Fig. 6). Chemical analysis of seven whole *G. aureomarginatus* individuals was carried out and resulted in the isolation and characterisation of dictyodendrins C (**1**) and F (**3**). The spectroscopic data (Table S1 to S6) were a close match to previously reported data (Kang et al. 2020; Warabi et al. 2003; Zhang et al. 2012a, b). The preference of *C. amoenum* for *D. teawanui* triggered our interest in this recently reported sponge species and chemical analysis yielded the known sterol, ergosterol peroxide, 5α,8α-epidioxy-24-methylcholesta-6,22-dien-3β-ol (**7**). The spectroscopic data (Table S7) were a close match to previously reported data (Gauvin et al. 2000). Several *C. amoenum* nudibranchs were extracted and the crude extract was purified by reversed phase C18 chromatography. Traces of ergosterol peroxide were detected by ^1^ H NMR spectroscopy in several fractions from chromatography of the nudibranchs but the compound was not detected in sufficient concentration to be characterised.

## Discussion

The feeding-choice experiments showed the preferences of two nudibranch species towards two different sponge species. Results obtained when only the first choice a nudibranch made was considered, and when all choices were considered, were similar for both nudibranch species. Neither sponge was significantly preferred and neither nudibranch was repulsed by either sponge before contact with the prey. Nudibranchs use their rhinophores as chemosensory receptors to detect olfactory clues in the water to find prey (Murphy et al. 1997). The feeding-choice experiments here utilized a no-flow set-up and therefore did not favour preliminary prey detection. Therefore, the results expected, if neither nudibranch species is repulsed by either sponge species, would be that each sponge would be chosen a similar number of times, which is what is shown in our results.

When the total time each nudibranch specimen spent grazing on either sponge was considered, results were significantly different. *G. aureonarginatus* specimens had an extremely clear preference for the Dictyodendrillid sponge. These results match observations in the wild, where half of the specimens collected were found on the Dictyodendrillid sponge and the remaining specimens were found crawling on the seafloor as opposed to on an alternative prey species. During the feeding-choice experiments, once the nudibranchs found the Dictyodendrillid sponge, they either stayed feeding on it for several hours and then continued crawling around the tank or stayed on the sponge until the end of the experiment. Nudibranchs are grazing organisms and considering the long time the specimens spent on the Dictyodendrillid sponge, one interpretation of the results is that the specimens left the Dictyodendrillid sponge when they were satiated. For five out of the six *G. aureomarginatus* specimens, each time *D. teawanui* was chosen, the nudibranchs crawled through the sponge but very quickly abandoned it. As these animals are slow-moving, the recorded time spent on *D. teawanui* is the time needed to crawl through the piece of sponge each time *D. teawanui* was found. *G. aureomarginatus* seems to have no specific interest in grazing on *D. teawanui*. Considering the difference in the time spent on either prey being particularly statistically significant, it indicates that *G. aureomarginatus* seems to be a specific predator. However, feeding-choice experiments with more sponge species should be conducted to confirm this. To date, *G. aureomarginatus* have only been reported grazing on *Dysidea fragilis (*Miller 1967), but no *Dysidea fragilis* have been recorded at the collection site.

When the total time each *C. amoenum* specimen spent grazing on either prey was considered, *D. teawanui* was the preferred sponge species, although to a lesser extent than *G. aureomarginatus* for the Dictyodendrillid sponge. Out of the four *C. amoenum* specimens studied, three had a significant preference for the blue *D. teawanui* sponge. The effects of grazing of *C. amoenum* on *D. teawanui* were clearly visible through comparison of pieces of sponge before and after some of the experiments. Once the specimens found *D. teawanui*, in a similar way to *G. auremarginatus*, they stayed for several hours grazing on it. *C. amoenum* is known to predate on *D. teawanui* (Mc Cormack et al. 2020) and the results reported here match observations in the wild where most of the specimens collected were found on *D. teawanui*. Even though *D. teawanui* appears to be the favourite prey of *C. amoenum*, the idea that it might be a generalist predator cannot be excluded given that *C. amoenum* is widely distributed across Australasian waters and has previously been reported grazing on *Dysidea fragilis* (Miller 1967). Considering our feeding-choice results and the previous report of the nudibranch on *D. fragilis*, it appears that *C. amoenum* may prefer to feed on the *Dysidea* genus but is not limited to it. Chemical studies of *Dysidea teawanui* are ongoing.

As stated above, the strong preference of *G. aureomarginatus* for the undescribed Dictyodendrillid sponge raised our interest in this unusual sponge. Chemical analysis of the sponge yielded the six known compounds dictyodendrins C (**1**), D (**2**) and F (**3**), as well as denigrin E (**4**), dactypyrrole A (**5**) and lamellarin O1 (**6**). Both dictyodendrin C (**1**) and D (**2**) have been reported from *Dictyodendrilla verongiformis*, a Japanese marine sponge (Warabi et al. 2003). **1** and **2** are structurally very similar, only differing by the presence of either one or two sodium sulfate groups. Further dictyodendrins (A, B and E) were reported from the same sponge (Warabi et al. 2003). Dictyodendrins A-E showed 100% inhibition of telomerase activity at a concentration of 50 μg/mL and were reported to be the first telomerase-inhibitory marine natural products. Dictyodendrin F (**3**), along with dictyodendrins G-J, were reported ten years later from a southern Australian marine sponge, *Ianthella* sp. (Zhang et al. 2012a, b) and from a *Dactylia* sp. marine sponge (Kang et al. 2020). Dictyodendrin F (**3**) was reported as the acid hydrolysis product of dictyodendrin A-E (Warabi et al. 2003) but was reported as a natural product from the *Ianthella* sp. sponge (Zhang et al. 2012a, b). Lamellarin O1 (7), along with lamellarin O and O2 have also been reported from an Australian *Ianthella* sp. marine sponge (Zhang et al. 2012a, b). Lamellarin alkaloids feature a 3,4-diarylpyrrole system and have been reported from diverse marine organisms, mainly ascidians (Carroll et al. 1993; Davis et al. 1999; Lindquist et al. 1988; Malla Reddy et al. 2005) and sponge (Urban et al. 1994, 1995). Some of these alkaloids are potently cytotoxic to various tumour cell lines (Pla et al. 2008). Denigrin E (**4**) and dactypyrrole A (**5**), along with denigrin D, F, G and dactylpyrrole B-C have been reported from a *Dactylia* marine sponge (Kang et al. 2020) and denigrin A-C have been isolated from the sponge *Dendrilla nigra*. (Kumar et al. 2014; Zhang et al. 2012a, b)

Chemical analysis of seven whole *G. aureomarginatus* individuals was carried out and resulted in the isolation and characterisation of dictyodendrins C (**1**) and F (**3**). This chemical link further establishes the relationship between the *G. aureomarginatus* nudibranchs and the Dictyodendrillid sponge. Dictyodendrins belong to a rare class of marine alkaloids and prior to our work, were only reported from two marine sponge species (Warabi et al. 2003; Zhang et al. 2012a, b) and had never been reported from mollusks. Due to the whole-body extraction, whether both dictyodendrins are located in specific parts of the nudibranch’s body is yet to be determined. Moreover, work is ongoing to determine if these two compounds are specifically being sequestered (e.g. for defence purposes) or just ingested while feeding and detected due to their higher concentration compared to other metabolites. Further taxonomic examination is needed to fully assign the Dictyodendrillid sponge to a species. It has not been found in New Zealand previously, and initial assessment suggests that it is a *Dicytodendrilla* genus, with skeletal similarities to *Dictyodendrilla tenella* (Lendenfled 1888) that has been recorded from Sydney Harbour (Atlas of Living Australia; World Register of Marine Species).

*C. amoenum*’s preference for *D. teawanui* triggered our interest in this recently reported sponge species and chemical analysis yielded the known sterol ergosterol peroxide, 5α,8α-epidioxy-24-methylcholesta-6,22-dien-3β-ol (**7**) (Gauvin et al. 2000). Ergosterol peroxide has been described from several sponge species including *Lendenfeldia chondrodes* (Sera et al. 1999), *Luffariella cf. variabilis* (Gauvin et al. 2000), and *Monanchora sp.* (Mun et al. 2015), and has been reported to possess growth inhibiting activity against various human cancer cells (Mun et al. 2015).

Establishing predator-prey relationships in the marine environment is of great ecological relevance and our results confirm that feeding-choice experiments in dorid nudibranchs are an efficient way to assess preferential prey. Both studied species have a preferred diet, with *G. aureomarginatus* potentially having an almost exclusive diet of the Dictyodendrillid sponge and *C. amoenum* showing preference for *D. teawanui*. The no-flow set-up allows best results when the time spent on prey is considered, instead of only considering the number of times a prey is chosen. Chemical analysis of both prey resulted in the isolation and characterisation of seven known natural compounds and two of them were also found in one of the nudibranch species, further establishing the predator-prey relationship. These feeding-choice experiments can both increase our knowledge of the dietary preferences and feeding habits of nudibranchs and potentially assist in the discovery of bioactive marine natural products.

### Electronic Supplementary Material

Below is the link to the electronic supplementary material.

Supplementary Material 1
