# Supplementary material for: Investigation of the Dietary Preferences of Two Dorid Nudibranchs by Feeding-Choice Experiments and Chemical Analysis
Source: J Chem Ecol. 2023 Jul 17;49(9-10):599–610. doi: 10.1007/s10886-023-01444-z (PMC10725399; doi:10.1007/s10886-023-01444-z)
Supplement: Supplementary file 1 — Supplementary Material 1 [file 10886_2023_1444_MOESM1_ESM.docx]

**Electronic Supplementary Information**

Journal of Chemical Ecology

Investigation of the dietary preferences of two dorid nudibranchs by feeding-choice experiments and chemical analysis

LAUREN GRIS^1^, CHRISTOPHER N. BATTERSHILL^2^ and MICHELE R. PRINSEP^1*^

*^1^Chemistry and Applied Physics, School of Science, University of Waikato, Private Bag 3105, Hamilton 3240, New Zealand.*

*^2^University of Waikato Coastal Marine Field Station, 58 Cross Road, Sulphur Point, Tauranga 3110, New Zealand.*

^*^corresponding author: [michele.prinsep@waikato.ac.nz](mailto:michele.prinsep@waikato.ac.nz)

**Table S1.** ^1^H and ^13^C NMR data for dictyodendrin C (**1**)

**Table S2.** ^1^H and ^13^C NMR data for dictyodendrin D (**2**)

**Table S3.** ^1^H NMR data for dictyodendrin F (**3**)

**Table S4.** ^1^H NMR data for denigrin E (**4**)

**Table S5.** ^1^H NMR data for dactypyrrole A (**5**)

**Table S6.** ^1^H and ^13^C NMR data for lamellarin O1 (**6**)

**Table S7.** ^1^H and ^13^C NMR data for 5α,8α-epidioxy-24-methylcholesta-6, 22-dien-3β-ol (**7**)

**Table S8.** *G. aureomarginatus* feeding-choice experiment with two sponge species as offered prey. Time spent on each prey (h) per specimen per individual experiment.

**Table S9.** *C. amoenum* feeding-choice experiment with two sponge species as offered prey. Time spent on each prey (h) per specimen per individual experiment.

**Figure S1a.** ESI(+)MS spectrum of dictyodendrin C (**1**)

**Figure S1b.** ESI(-)MS spectrum of dictyodendrin C (**1**)

**Figure S2a.** ESI(+)MS spectrum of dictyodendrin D (**2**)

**Figure S2b.** ESI(-)MS spectrum of dictyodendrin D (**2**)

**Figure S3a.** ESI(+)MS spectrum of dictyodendrin F (**3**)

**Figure S3b.** ESI(-)MS spectrum of dictyodendrin F (**3**)

**Figure S4a.** ESI(+)MS spectrum of denigrin E (**4**)

**Figure S4b.** ESI(-)MS spectrum of denigrin E (**4**)

**Figure S5a.** ESI(+)MS spectrum of dactypyrrole A (**5**)

**Figure S5b.** ESI(-)MS spectrum of dactypyrrole A (**5**)

**Figure S6a.** ESI(+)MS spectrum of lamellarin O1 (**6**)

**Figure S6b.** ESI(-)MS spectrum of lamellarin O1 (**6**)

**Figure S7.** ESI(+)MS spectrum of 5α,8α-epidioxy-24-methylcholesta-6, 22-dien-3β-ol (**7**).


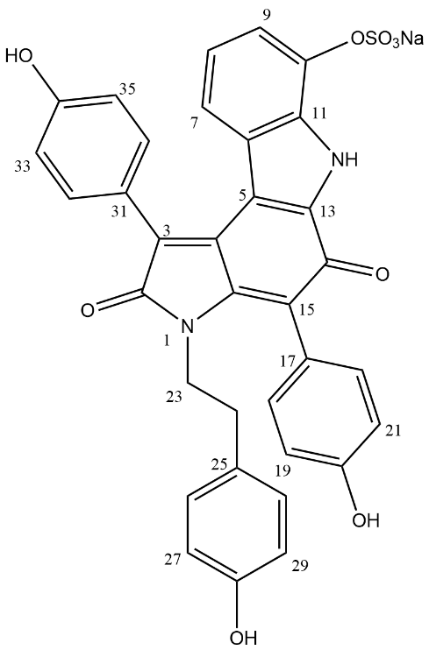


dictyodendrin C

**Table S1.** ^1^H and ^13^C NMR data for dictyodendrin C (**1**)

|  | **^1^H** δ_H_ (mult, *J* in Hz) | | | **^13^C** δ_C_ | |
| --- | --- | --- | --- | --- | --- |
| **Atom** | **Experimental^a^** | | **Literature^b^** | **Experimental^a^** | **Literature^b^** |
|  | Dictyodendrillid sponge | *G.aureomarginatus* |  | Dictyodendrillid sponge |  |
| **2** |  |  |  | 173.3 | 173.2 |
| **3** |  |  |  | 130.4 | 130.4 |
| **4** |  |  |  | 135.3 | 135.2 |
| **5** |  |  |  | 114.0 | 114.0 |
| **6** |  |  |  | 126.7 | 126.8 |
| **7** | 6.13 (1H, d, 8.3) | 6.13 (1H, d, 7.5) | 6.13 (1H, d, 8.1) | 121.8 | 121.8 |
| **8** | 6.70 (1H, dd, 8.0, 8.0) | 6.70 (1H, dd, 8.1, 8.0) | 6.70 (1H, dd, 8.1, 8.1) | 122.4 | 122.3 |
| **9** | 7.19 (1H, d, 7.7) | 7.19 (1H, d, 7.8) | 7.19 (1H, d, 8.1) | 118.2 | 118.2 |
| **10** |  |  |  | 140.7 | 140.6 |
| **11** |  |  |  | 133.4 | 133.4 |
| **13** |  |  |  | 133.7 | ND^g^ |
| **14** |  |  |  | 180.7 | 180.7 |
| **15** |  |  |  | 119.4 | 129.4^d^ |
| **16** |  |  |  | 150.4 | 150.3 |
| **17** |  |  |  | 124.1 | 124.0 |
| **18/22** | 7.23 (2H, d, 8.5) | 7.23 (2H, d, 8.6) | 7.22 (2H, d, 8.6) | 133.8^e^ | 133.4 |
| **19/21** | 6.91 (2H, d, 8.5) | 6.91 (2H, d, 8.6) | 6.90 (2H, d, 8.6) | 116.1^f^ | 116.0 |
| **20** |  |  |  | 159.2 | 159.3 |
| **23** | 3.41 (2H, t, 7.8) | ^c^ | 3.40 (2H, t, 8.1) | 44.2 | 46.1 |
| **24** | 2.38 (2H, t, 8.0) | 2.41 (2H, t, 7.9) | 2.73 (2H, t, 8.1) | 34.9 | 34.9 |
| **25** |  |  |  | 130.0 | 130.0 |
| **26/30** | 6.63 (2H, d, 8.4) | 6.64 (2H, d, 8.5) | 6.63 (2H, d, 8.4) | 130.9 | 130.9 |
| **27/29** | 6.54 (2H, d, 8.4) | 6.55 (2H, d, 8.5) | 6.55 (2H, d, 8.4) | 116.0^f^ | 116.0 |
| **28** |  |  |  | 156.9 | 157.0 |
| **31** |  |  |  | 123.7 | 123.6 |
| **32/36** | 7.29 (2H, d, 8.5) | 7.30 (2H, d, 8.6) | 7.30 (2H, d, 8.6) | 133.5^e^ | 133.4 |
| **33/35** | 6.88 (2H, d, 8.5) | 6.88 (2H, d, 8.7) | 6.88 (2H, d, 8.6) | 116.4 | 116.4 |
| **34** |  |  |  | 160.3 | 160.3 |

^a^ values reported in CD_3_OD, ^1^H 600 MHz, ^13^C 150 MHz ^b^ values reported in CD_3_OD, ^1^H 600 MHz, ^13^C 150 MHz (Warabi et al. 2003) ^c^ undetermined due to overlapping signals
^d^ the value reported appears downfield compared to the carbon in position 15, reported at 119.3 ppm, for the closely related dictyodendrin D
(Warabi et al. 2003). It could be due to a typographical error.
^e^ assignment may be interchanged
^f^ assignment may be interchanged
^g^ not determined


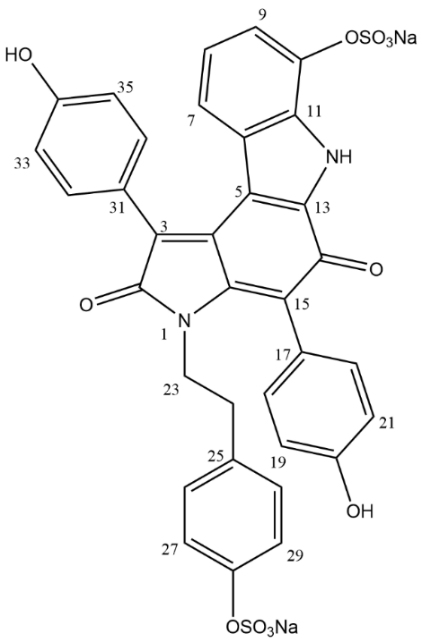


dictyodendrin D

**Table S2.** ^1^H and ^13^C NMR data for dictyodendrin D (**2**)

|  | **^1^H** δ_H_ (mult, *J* in Hz) | | **^13^C** δ_C_ | |
| --- | --- | --- | --- | --- |
| **Atom** | **Experimental^a^** | **Literature^b^** | **Experimental^a^** | **Literature^b^** |
| **2** |  |  | 173.2 | 173.2 |
| **3** |  |  | 130.4 | 130.5 |
| **4** |  |  | 135.2 | 135.0 |
| **5** |  |  | 114.0 | 114.0 |
| **6** |  |  | 126.7 | 126.8 |
| **7** | 6.15 (1H, d, 8.2) | 6.15 (1H, d, 8.1) | 121.9 | 121.9 |
| **8** | 6.72 (1H, dd, 8.1, 7.9) | 6.72 (1H, dd, 8.1, 8.1) | 122.4 | 122.4 |
| **9** | 7.19 (1H, d, 7.8) | 7.20 (1H, d, 8.1) | 118.2 | 118.2 |
| **10** |  |  | 140.8 | 140.7 |
| **11** |  |  | 133.4 | 133.5 |
| **13** |  |  | 133.7 | 133.7 |
| **14** |  |  | 180.8 | 180.6 |
| **15** |  |  | 119.4 | 119.3 |
| **16** |  |  | 150.1 | 150.2 |
| **17** |  |  | 124.0 | 124.0 |
| **18/22** | 7.26 (2H, d, 8.6) | 7.24 (2H, d, 8.6) | 133.8 | 133.8 |
| **19/21** | 6.92 (2H, d, 8.6) | 6.92 (2H, d, 8.6) | 116.2 | 116.3 |
| **20** |  |  | 159.3 | 158.8 |
| **23** | 3.44 (2H, t, 8.2) | 3.44 (2H, t, 8.1) | 43.9 | 44.0 |
| **24** | 2.49 (2H, t, 8.2) | 2.48 (2H, t, 8.1) | 35.2 | 35.1 |
| **25** |  |  | 135.8 | 135.8 |
| **26/30** | 6.76 (2H, d, 8.6) | 6.76 (2H, d, 8.6) | 130.5 | 130.5 |
| **27/29** | 7.09 (2H, d, 8.6) | 7.04 (2H, d, 8.6) | 122.4 | 122.3 |
| **28** |  |  | 152.5 | 152.8 |
| **31** |  |  | 123.7 | 123.7 |
| **32/36** | 7.35 (2H, d, 8.8) | 7.34 (2H, d, 8.6) | 133.5 | 133.5 |
| **33/35** | 6.90 (2H, d, 8.8) | 6.90 (2H, d, 8.6) | 116.4 | 116.5 |
| **34** |  |  | 160.4 | 160.6 |

^a^ values reported in CD_3_OD, ^1^H 600 MHz, ^13^C 150 MHz ^b^ values reported in CD_3_OD, ^1^H 600 MHz, ^13^C 150 MHz (Warabi et al. 2003)


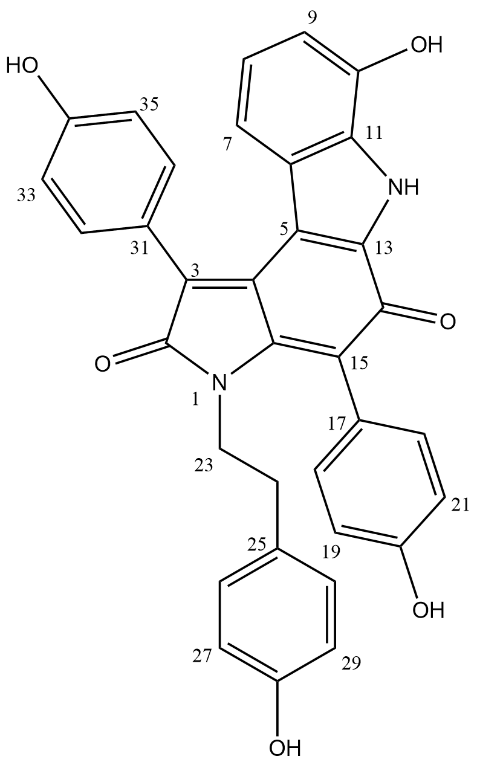


dictyodendrin F

**Table S3.** ^1^H NMR data for dictyodendrin F (**3**)

|  | **^1^H** δ_H_ (mult, J in Hz) | | | |
| --- | --- | --- | --- | --- |
| **Atom** | **Experimental^a^** | | **Literature^b^** | **Literature^c^** |
|  | Dictyodendrillid sponge | *G.aureomarginatus* |  |  |
| **7** | 5.83 (dd, 7.3, 1.6) | 5.83 (dd, 7.7, 1.5) | 5.83 (dd, 7.8, 1.2) | 5.83 (dd, 7.3, 1.9) |
| **8** | 6.56-6.59^d^ | 6.56-6.59^d^ | 6.55-6.60^d^ | 6.58 (t, 7.3) |
| **9** | 6.56-6.59^d^ | 6.56-6.59^d^ | 6.55-6.60^d^ | 6.57 (dd, 7.3, 1.9) |
| **18/22** | 7.24 (d, 8.3) | 7.26 (d, 8.5) | 7.25 (d, 8.6) | 7.24 (d, 8.5) |
| **19/21** | 6.93 (d, 8.3) | 6.94 (d, 8.6) | 6.94 (d, 8.6) | 6.93 (d, 8.6) |
| **23** | 3.42 (t, 8.0) | ^d^ | 3.43 (t, 8.1) | 3.42 (t, 7.8) |
| **24** | 2.40 (t, 8.0) | 2.42 (t, 7.9) | 2.41 (t, 8.1) | 2.41 (t, 8.1) |
| **26/30** | 6.56-6.59^d^ | 6.56-6.59^d^ | 6.55-6.60^d^ | 6.56 (d, 8.5) |
| **27/29** | 6.65 (d, 8.3) | 6.67 (d, 8.5) | 6.66 (d, 8.5) | 6.66 (d, 8.5) |
| **32/36** | 7.31 (d, 8.5) | 7.32 (d, 8.6) | 7.32 (d, 8.6) | 7.31 (d, 8.5) |
| **33/35** | 6.92 (d, 8.5) | 6.90 (d, 8.6) | 6.90 (d, 8.6) | 6.89 (d, 8.5) |

^a^ values reported in CD_3_OD, ^1^H 600 MHz,
^b^ values reported in CD_3_OD, ^1^H 600 MHz. ^13^C assignment only reported in pyridine-d5. Dictyodendrin F reported as a natural product (Zhang et al. 2012).
^c^ values reported in CD_3_OD, 600 MHz. ^13^C assignment only reported in pyridine-d5. Dictyodendrin F reported as acid hydrolysis product (Warabi et al. 2003).
^d^ overlapping signals


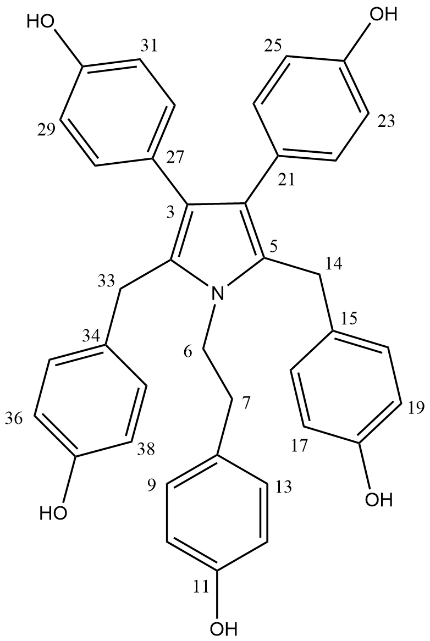


denigrin E

**Table S4.** ^1^H NMR data for denigrin E (**4**)

|  | **^1^H** δ_H_ (mult, *J* in Hz) | |
| --- | --- | --- |
| **Atom** | **Experimental^a^** | **Literature^b^** |
| **6** | 3.57 (2H, t) | 3.57 (2H, t) |
| **7** | 2.43 (2H, t) | 2.43 (2H, t) |
| **9/13** | 6.61-6.93^c^ | 6.63-6.95^c^ |
| **10/12** | 6.61-6.93^c^ | 6.63-6.95^c^ |
| **14/33** | 3.81 (s, 4H) | 3.82 (4H, s) |
| **16/20/35/39** | 6.61-6.93^c^ | 6.63-6.95^c^ |
| **17/19/36/38** | 6.61-6.93^c^ | 6.63-6.95^c^ |
| **22/26/28/32** | 6.61-6.93^c^ | 6.63-6.95^c^ |
| **23/25/29/31** | 6.61-6.93^c^ | 6.63-6.95^c^ |

^a^ values reported in CD_3_OD, 600 MHz ^b^ values reported in CD_3_OD, 600 MHz. ^1^H and ^13^C assignment reported in pyridine-d5. Only ^1^H spectra reported in CD_3_OD (Kang et al. 2020)
^c^ unassigned due to overlapping signals and literature data unreported in CD_3_OD.

dactylpyrrole A


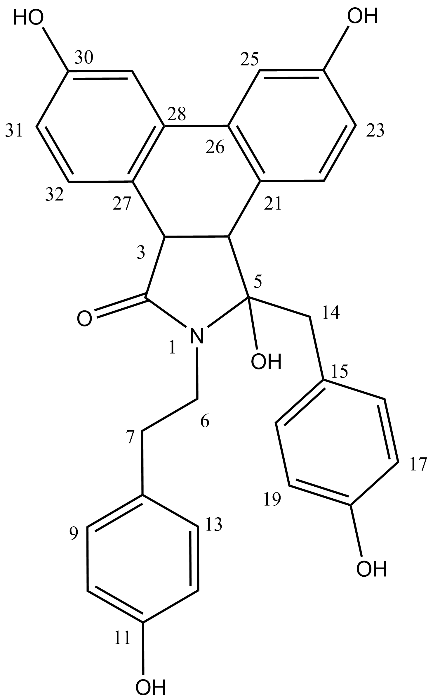


**Table S5.** ^1^H NMR data for dactypyrrole A (**5**)

|  | **^1^H** δ_H_ (mult, *J* in Hz) | |
| --- | --- | --- |
| **Atom** | **Experimental^a^** | **Literature^b^** |
| **6** | 3.62 (m^c^) 3.85 (ddd, 13.8, 12.2, 5.4) | 3.62 (m^c^) 3.85 (ddd, 13.6, 11.9, 5.2) |
| **7** | 2.92 (dt, 12.3, 5.1) 3.08 (td, 12.3, 5.1) | 2.93 (ddd, 12.7, 12.5, 5.3) 3.08 (ddd, 12.4, 12.3, 5.1) |
| **9/13** | 7.22 (d, 8.4) | 7.22 (d, 8.4) |
| **10/12** | 6.78 (d, 8.4) | 6.78 (d, 8.4) |
| **14** | 3.50 (d, 14.1) 3.62 (m^c^) | 3.51 (d, 14.1) 3.62 (m^c^) |
| **16/19** | 6.25 (d, 8.7) | 6.25 (d, 8.7) |
| **17/20** | 6.21 (d, 8.7) | 6.21 (d, 8.7) |
| **22** | 8.50 (d, 8.8) | 8.50 (d, 8.8) |
| **23** | 7.33 (dd, 8.8, 2.4) | 7.34 (dd, 8.8, 2.4) |
| **25** | 8.01 (d, 2.4) | 8.02 (d, 2.4) |
| **29** | 7.91 (d, 2.4) | 7.92 (d, 2.4) |
| **31** | 7.11 (dd, 8.8, 2.4) | 7.12 (dd, 8.8, 2.4) |
| **32** | 8.83 (d, 8.8) | 8.84 (d, 8.8) |

^a^ values reported in CD_3_OD, 600 MHz^b^ values reported in CD_3_OD, 600 MHz (Kang et al. 2020)
^c^ overlapping signals


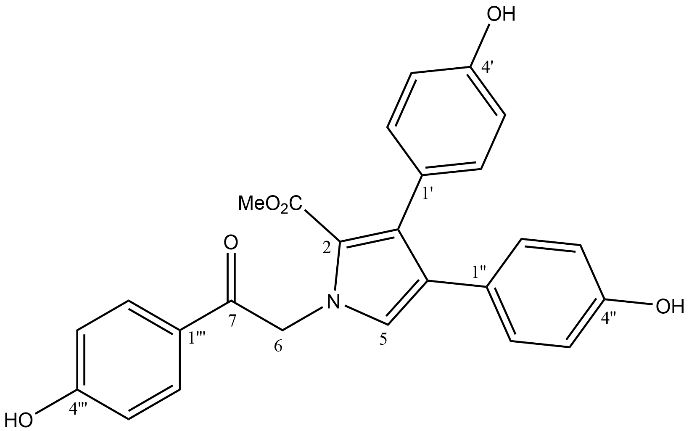


lamellarin O1

**Table S6.** ^1^H NMR data for lamellarin O1 (**6**)

|  | **^1^H** δ_H_ (mult, *J* in Hz) | | **^13^C** δ_C_ | |
| --- | --- | --- | --- | --- |
| **Atom** | **Experimental^a^** | **Literature^b^** | **Experimental^a^** | **Literature^b^** |
| **1’** |  |  | 128.6 | 128.8 |
| **2’/6’** | 6.99 (d, 8.5) | 7.00 (d, 8.6) | 132.9 | 133.1 |
| **3’/5’** | 6.70 (d, 8.6) | 6.70 (d, 8.6) | 115.3 | 115.5 |
| **4’** |  |  | 157.3 | 157.3 |
| **1’’** |  |  | 127.5 | 127.7 |
| **2’’/6’’** | 6.91 (d, 8.8) | 6.91 (d, 8.8) | 130.4 | 130.6 |
| **3’’/5’’** | 6.57 (d, 8.7) | 6.58 (d, 8.8) | 115.8 | 116.0 |
| **4’’** |  |  | 156.6 | 156.8 |
| **1’’** |  |  | 127.5 | 127.0 |
| **2’’’/6’’’** | 7.98 (d, 8.5) | 7.95 (d, 8.5) | 131.7 | 132.0 |
| **3’’’/5’’’** | 6.90 (d, 8.5) | 6.85 (d, 8.5) | 116.5 | 117.4 |
| **4’’’** |  |  | 163.9 | 167.1 |
| **2** |  |  | 120.8 | 121.0 |
| **2-CO_2_CH_3_** |  |  | 164.3 | 164.0 |
| **3** |  |  | 132.4 | 132.8 |
| **4** |  |  | 126.0 | 126.1 |
| **5** | 7.07 (s) | 7.07 (s) | 128.5 | 128.7 |
| **6** | 5.81 (s) | 5.81 (s) | 56.7 | 56.8 |
| **7** |  |  | 194.8 | 194.8 |
| **2-CO_2_CH_3_** | 3.42 (s) | 3.42 (s) | 50.9 | 51.1 |

^a^ values reported in CD_3_OD, 600 MHz ^b^ values reported in CD_3_OD, 600 MHz (Zhang et al. 2012)

5α,8α-epidioxy-24-methylcholesta-6, 22-dien-3β-ol


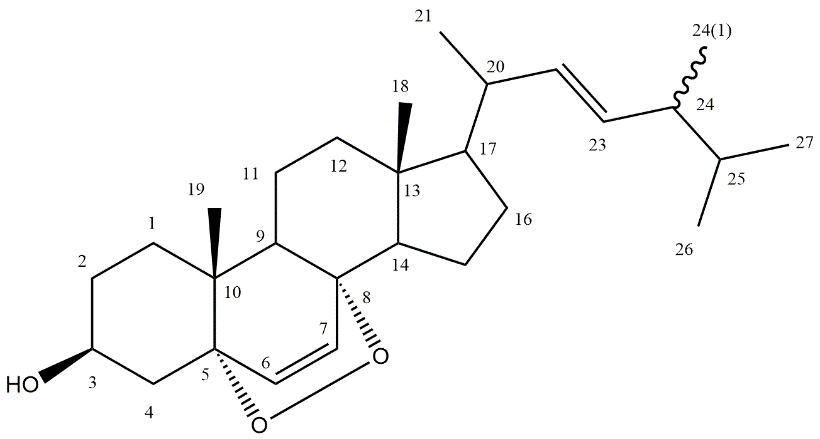


**Table S7.** ^1^H and ^13^C NMR data for 5α,8α-epidioxy-24-methylcholesta-6, 22-dien-3β-ol (**7**).

|  | **^1^H** δ_H_ (mult, *J* in Hz) | | **^13^C** δ_C_ | |
| --- | --- | --- | --- | --- |
| **Atom** | **Experimental^a^** | **Literature^b^** | **Experimental^a^** | **Literature^b^** |
| **1** |  |  | 34.83 | 34.83 |
| **2** |  |  | 30.27 | 30.26 |
| **3** | 3.97 (m) | 3.95 (m) | 66.65 | 66.58 |
| **4** |  |  | 37.09 | 37.08 |
| **5** |  |  | 82.30 | 82.24 |
| **6** | 6.24 (d, 8.3) | 6.22 (d, 8.5) | 135.56 | 135.52 |
| **7** | 6.51 (d, 8.3) | 6.48 (d, 8,5) | 130.93 | 130.83 |
| **8** |  |  | 79.61 | 79.52 |
| **9** |  |  | 51.22 | 51.25 |
| **10** |  |  | 37.08 | 37.08 |
| **11** |  |  | 23.56 | 23.52 |
| **12** |  |  | 39.58 | 39.56 |
| **13** |  |  | 44.71 | 44.68 |
| **14** |  |  | 51.84 | 51.84 |
| **15** |  |  | 20.76 | 20.78 |
| **16** |  |  | 28.88 | 28.98 |
| **17** |  |  | 56.57 | 56.31 |
| **18** | 0.80 (s) | 0.79 (s) | 13.00 | 12.96 |
| **19** | 0.88 (s) | 0.86 (s) | 18.31 | 18.27 |
| **20** |  |  | 39.95 | 39.87 |
| **21** | 0.97 (d, 6.2) | 0.97 (d, 6.6) | 21.06 | 21.02 |
| **22** | 5.19 (dd, 7.5, 15.2) | 5.17 (dd, 7.5, 15.2) | 135.54 | 135.52 |
| **23** | 5.13 (dd, 7.6, 15.2) | 5.11 (dd, 7.8, 15.2) | 132.57 | 132.56 |
| **24** |  |  | 43.20 | 43.15 |
| **24(1)** | 0.88 (^c^) | 0.89 (d, 6.8) | 18.17 | 18.27 |
| **25** | 0.81 (^c^) | 0.79 (d, 7.0) | 33.34 | 33.31 |
| **26** | 0.82 (^c^) | 0.81 (d, 7.2) | 20.30 | 20.22 |
| **27** |  |  | 19.80 | 19.75 |

^a^ values reported in CDCl_3_, ^1^H 600 MHz, ^13^C 150 MHz ^b^ values reported in CDCl_3_, ^1^H 400 MHz, ^13^C 100 MHz (Gauvin et al. 2000) ^c^ undetermined due to overlapping signals

**Table S8.** *G. aureomarginatus* feeding-choice experiment with two sponge species as offered prey. Time spent on each prey (h) per specimen per individual experiment.

|  |  | Specimen 1 | Specimen 2 | Specimen 3 | Specimen 4 | Specimen 5 | Specimen 6 |
| --- | --- | --- | --- | --- | --- | --- | --- |
| Experiment 1 | *D. teawanui* | 0.0 | 0.0 | 0.0 | 0.2 | 0.4 | 0.0 |
|  | Black sponge | 11.9 | 0.0 | 0.0 | 0.0 | 1.2 | 0.0 |
| Experiment 2 | *D. teawanui* | 0.0 | 0.5 | 0.0 | 0.0 | 0.0 | 0.1 |
|  | Black sponge | 0.4 | 0.9 | 0.4 | 0.0 | 0.0 | 0.0 |
| Experiment 3 | *D. teawanui* | 0.0 | 0.3 | 0.1 | 0.0 | 0.2 | 0.3 |
|  | Black sponge | 2.6 | 1.0 | 0.1 | 0.0 | 0.1 | 0.0 |
| Experiment 4 | *D. teawanui* | 0.0 | 0.0 | 0.0 | 0.0 | 0.2 | 0.0 |
|  | Black sponge | 0.1 | 9.1 | 0.0 | 0.0 | 0.0 | 0.0 |
| Experiment 5 | *D. teawanui* | 0.0 | 0.0 | 0.1 | 0.0 | 0.0 | 0.0 |
|  | Black sponge | 0.0 | 0.0 | 0.0 | 0.0 | 0.5 | 5.2 |
| Experiment 6 | *D. teawanui* | 0.0 | 0.0 | 0.0 | 0.0 | 0.0 | 0.0 |
|  | Black sponge | 0.0 | 5.9 | 12.5 | 0.0 | 0.0 | 0.0 |
| Experiment 7 | *D. teawanui* | 0.0 | 0.0 | 0.0 | 0.0 | 0.0 | 0.0 |
|  | Black sponge | 0.0 | 9.8 | 0.0 | 6.4 | 5.5 | 0.9 |
| Experiment 8 | *D. teawanui* | 0.0 | 0.0 | 0.0 | 0.0 | 0.0 | 0.0 |
|  | Black sponge | 0.9 | 0.0 | 0.0 | 0.0 | 0.0 | 2.4 |
| Experiment 9 | *D. teawanui* | 0.0 | 5.2 |  |  |  |  |
|  | Black sponge | 0.0 | 0.0 |  |  |  |  |
| Experiment 10 | *D. teawanui* | 0.0 | 0.0 |  |  |  |  |
|  | Black sponge | 0.4 | 4.9 |  |  |  |  |
| Experiment 11 | *D. teawanui* | 0.0 | 0.0 |  |  |  |  |
|  | Black sponge | 0.0 | 13.9 |  |  |  |  |
| Cumulative time spent on prey (h) | *D. teawanui* | 0.1 | 6.0 | 0.3 | 0.2 | 0.9 | 0.4 |
|  | Black sponge | 16.4 | 45.5 | 13.0 | 6.4 | 7.3 | 8.5 |

**Table S9.** *C. amoenum* feeding-choice experiment with two sponge species as offered prey. Time spent on each prey (h) per specimen per individual experiment.

|  |  | Specimen 1 | Specimen 2 | Specimen 3 | Specimen 4 |
| --- | --- | --- | --- | --- | --- |
| Experiment 1 | *D. teawanui* | 0.0 | 0.0 | 7.3 | 6.0 |
|  | Black sponge | 0.0 | 0.0 | 0.0 | 0.2 |
| Experiment 2 | *D. teawanui* | 0.0 | 0.2 | 0.0 | 0.0 |
|  | Black sponge | 0.0 | 0.0 | 2.9 | 0.0 |
| Experiment 3 | *D. teawanui* | 0.0 | 0.0 | 0.0 | 0.0 |
|  | Black sponge | 0.0 | 0.0 | 0.0 | 0.0 |
| Experiment 4 | *D. teawanui* | 0.0 | 9.5 | 0.0 | 0.0 |
|  | Black sponge | 0.0 | 0.3 | 0.0 | 0.0 |
| Experiment 5 | *D. teawanui* | 0.0 | 0.0 | 0.0 | 0.0 |
|  | Black sponge | 0.0 | 0.0 | 0.0 | 0.0 |
| Experiment 6 | *D. teawanui* | 10.5 | 15.3 | 0.0 | 11.2 |
|  | Black sponge | 0.0 | 0.0 | 0.0 | 0.0 |
| Experiment 7 | *D. teawanui* | 0.0 | 0.0 | 0.0 | 0.0 |
|  | Black sponge | 0.0 | 0.0 | 1.3 | 0.0 |
| Experiment 8 | *D. teawanui* | 0.0 | 0.2 | 0.0 | 0.0 |
|  | Black sponge | 0.0 | 0.0 | 0.0 | 0.0 |
| Experiment 9 | *D. teawanui* | 0.0 | 0.0 | 0.0 | 0.0 |
|  | Black sponge | 0.0 | 0.0 | 0.0 | 0.0 |
| Experiment 10 | *D. teawanui* | 0.0 | 0.0 | 0.0 | 0.0 |
|  | Black sponge | 2.3 | 0.0 | 0.0 | 0.0 |
| Experiment 11 | *D. teawanui* | 0.0 | 0.0 | 0.0 | 0.0 |
|  | Black sponge | 24.0 | 0.0 | 0.0 | 0.1 |
| Cumulative time spent on prey (h) | *D. teawanui* | 10.4 | 25.1 | 7.3 | 17.2 |
|  | Black sponge | 26.3 | 0.4 | 4.1 | 0.3 |

**Figure S1a.** ESI(+)MS spectrum of dictyodendrin C (**1**)

Frag = 150V


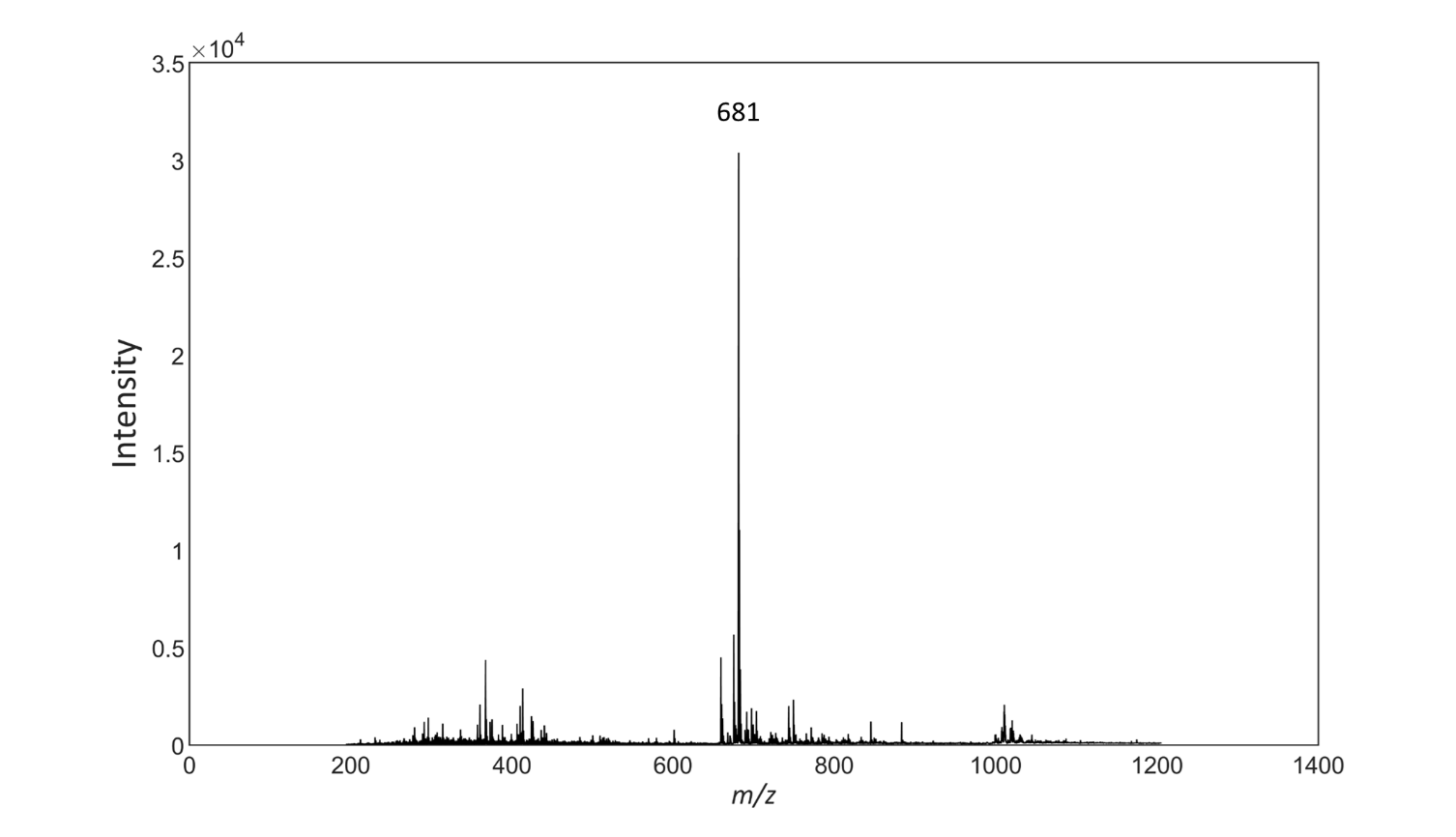


**Figure S1b.** ESI(-)MS spectrum of dictyodendrin C (**1**)


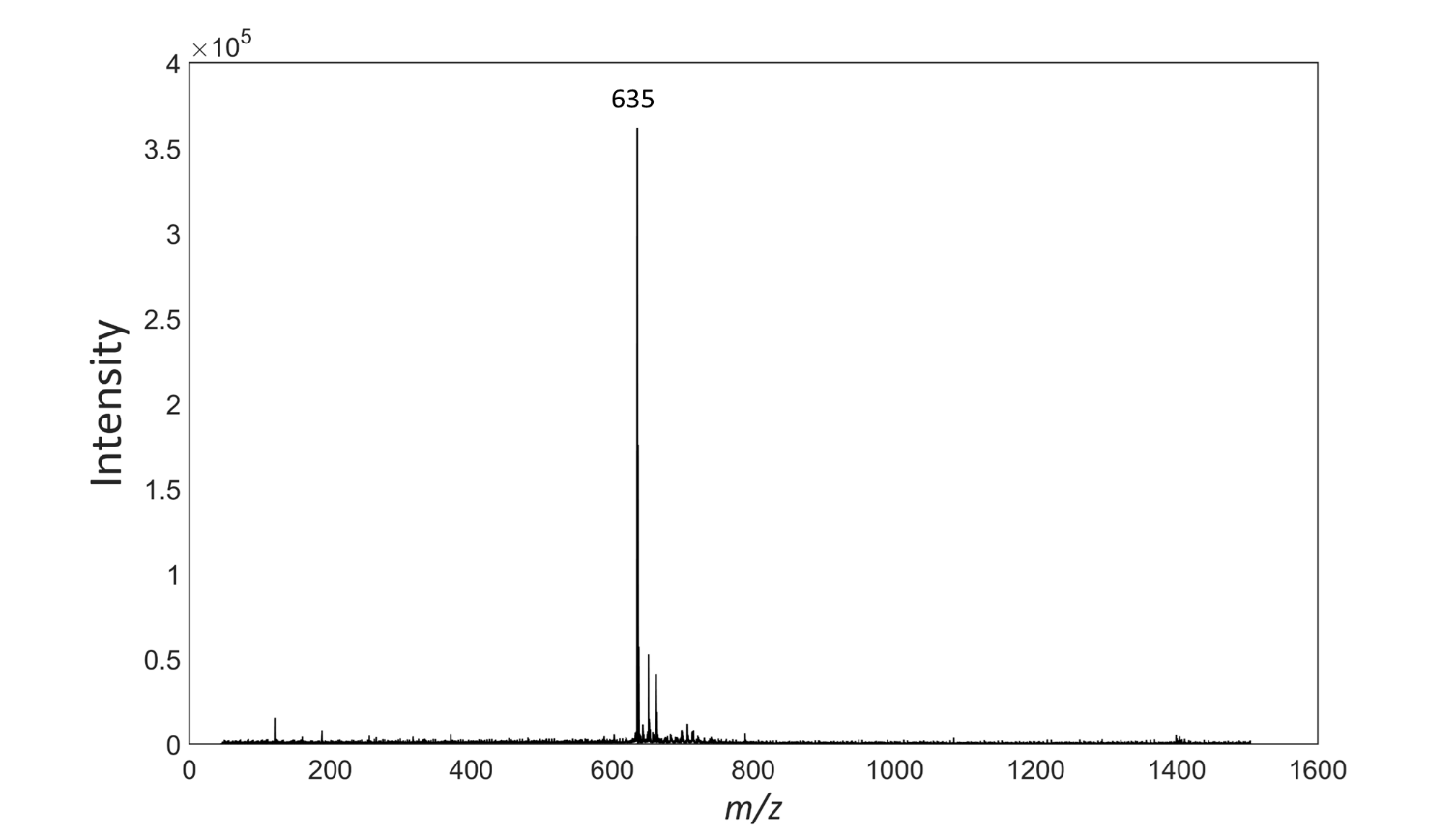


Frag = -150V

**Figure S2a.** ESI(+)MS spectrum of dictyodendrin D (**2**)


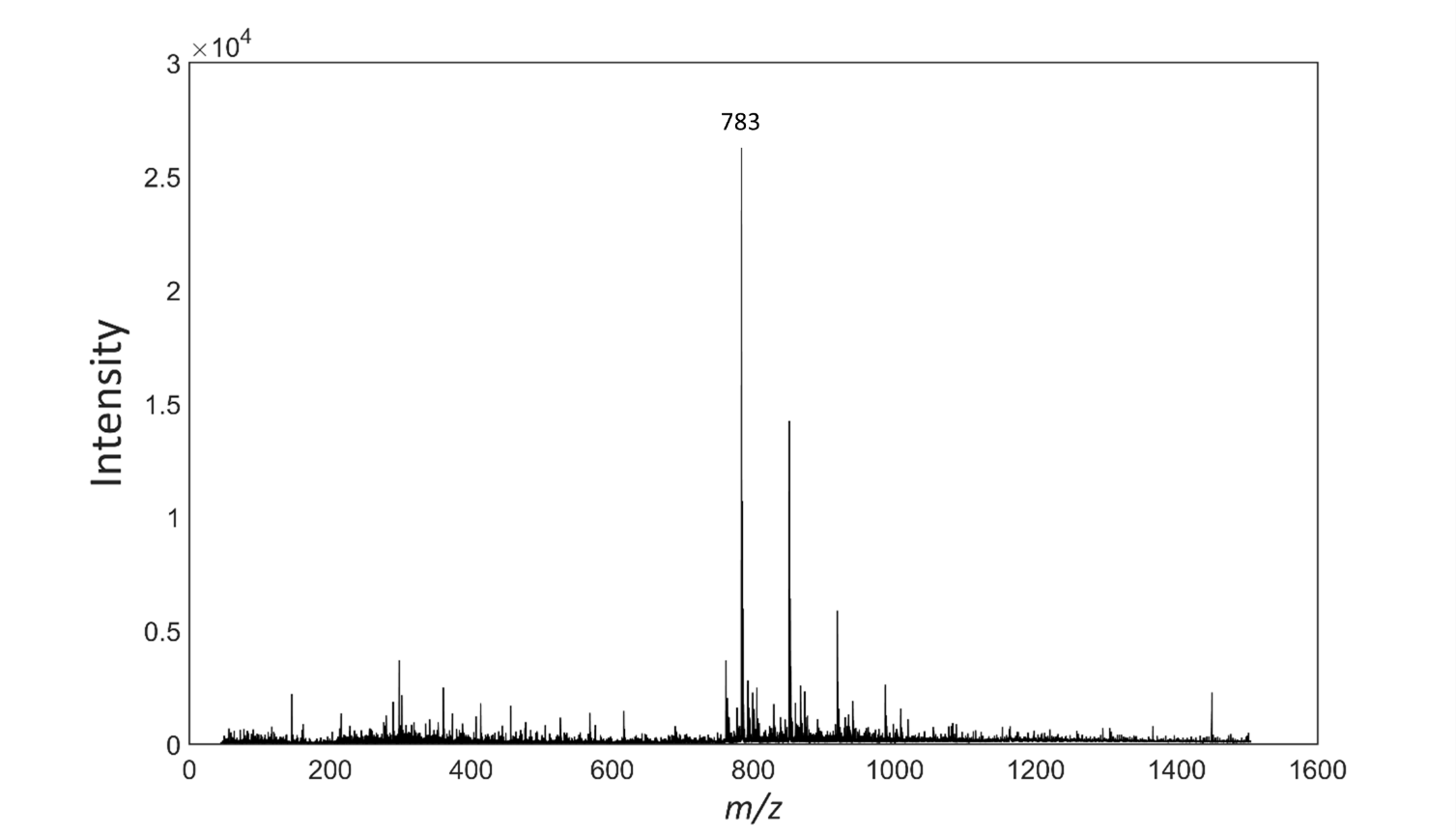


Frag = 150V

Frag = -90V

**Figure S2b.** ESI(-)MS spectrum of dictyodendrin D (**2**)


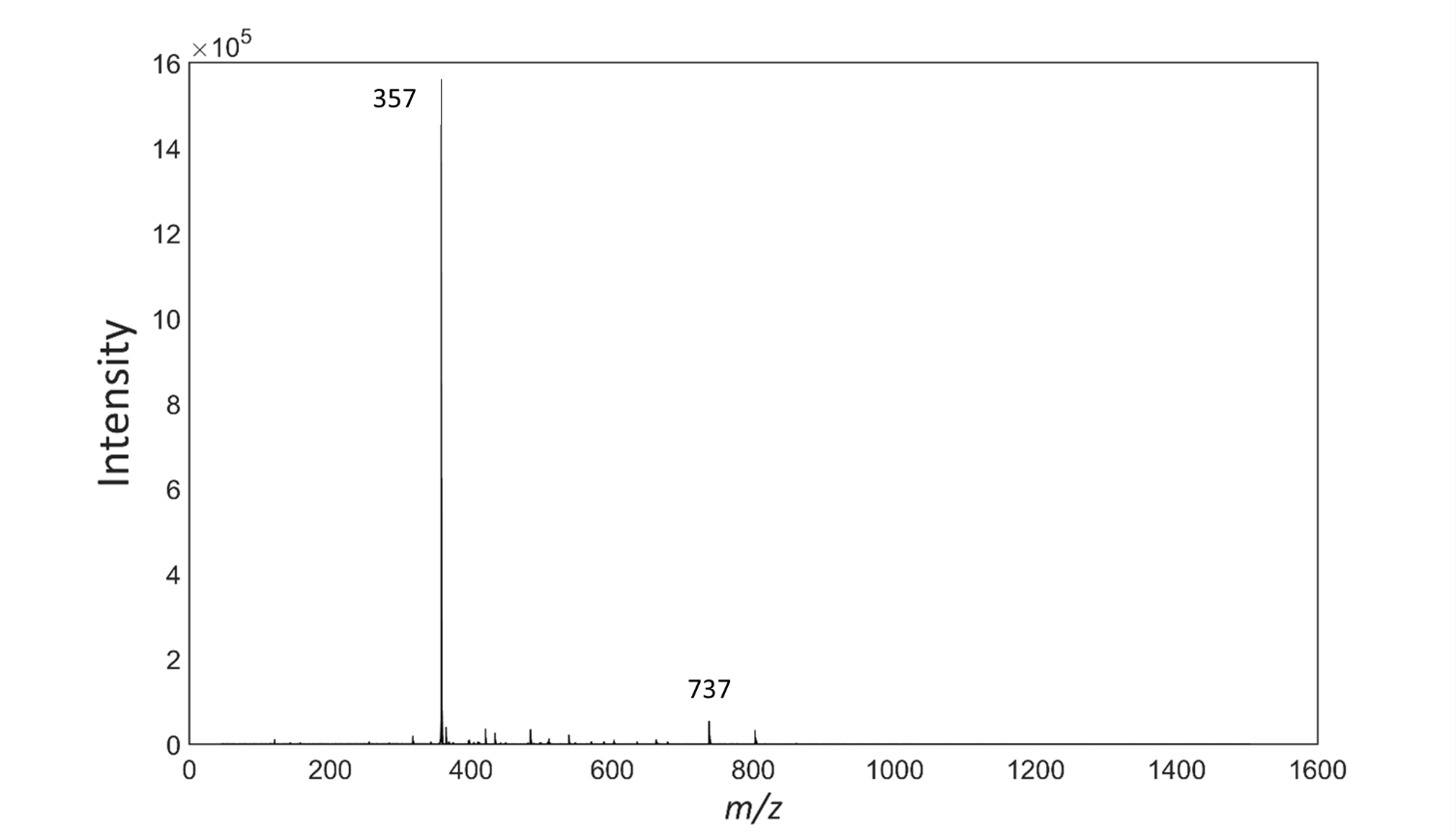


Frag = -90V

**Figure S3a.** ESI(+)MS spectrum of dictyodendrin F (**3**)


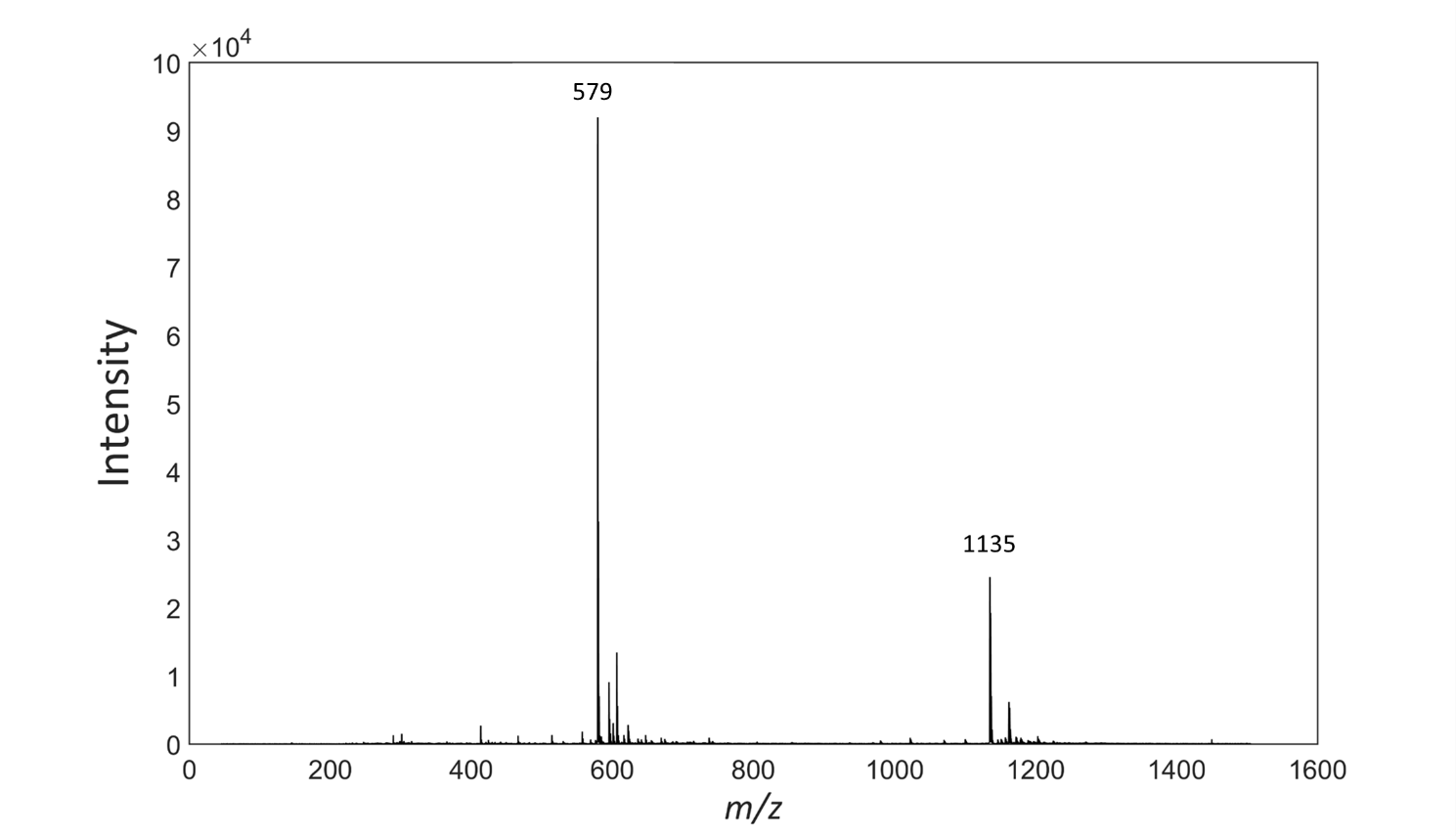


Frag = 150V

**Figure S3b.** ESI(-)MS spectrum of dictyodendrin F (**3**)


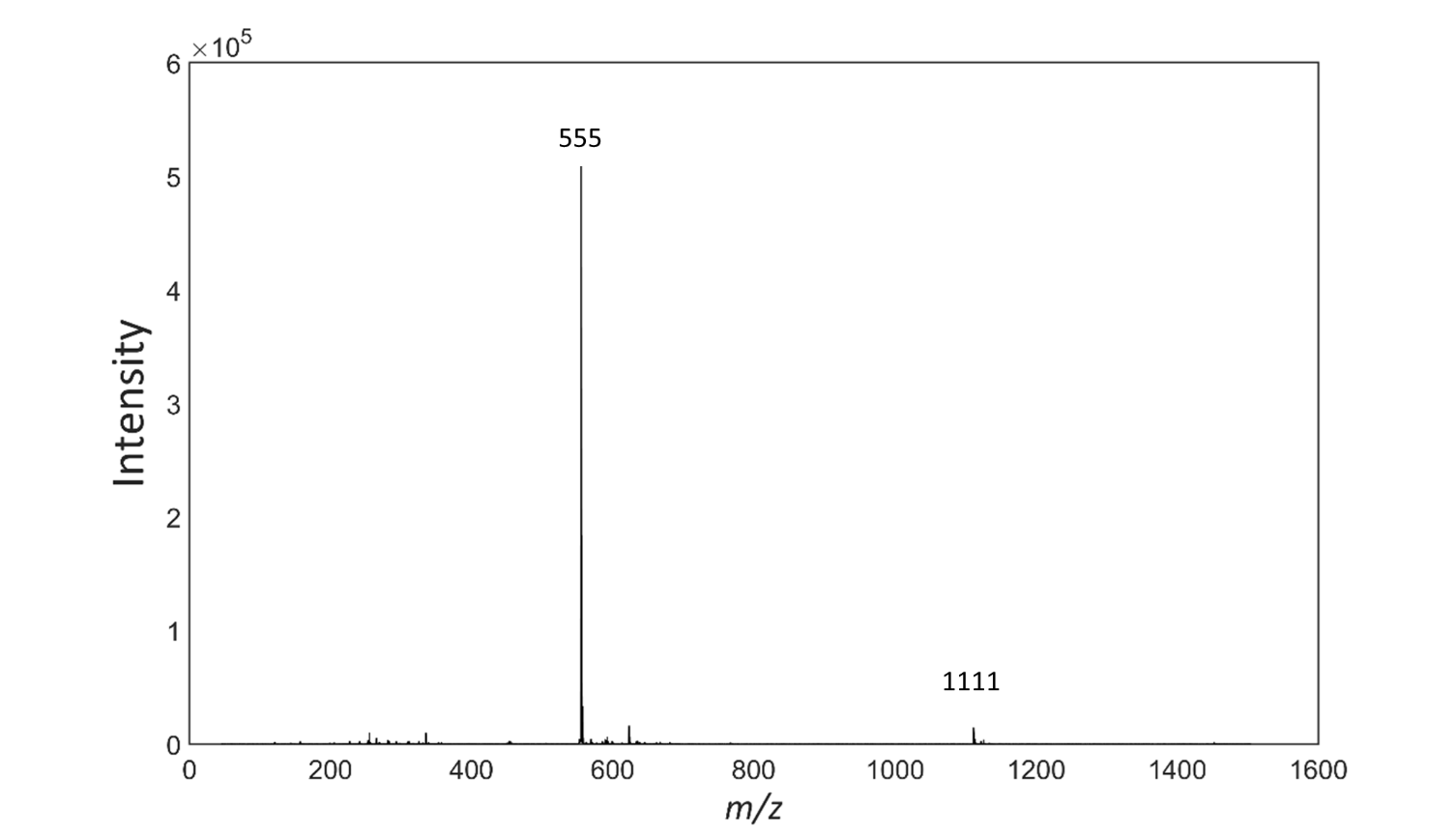


Frag = -150V

**Figure S4a.** ESI(+)MS spectrum of denigrin E (**4**)


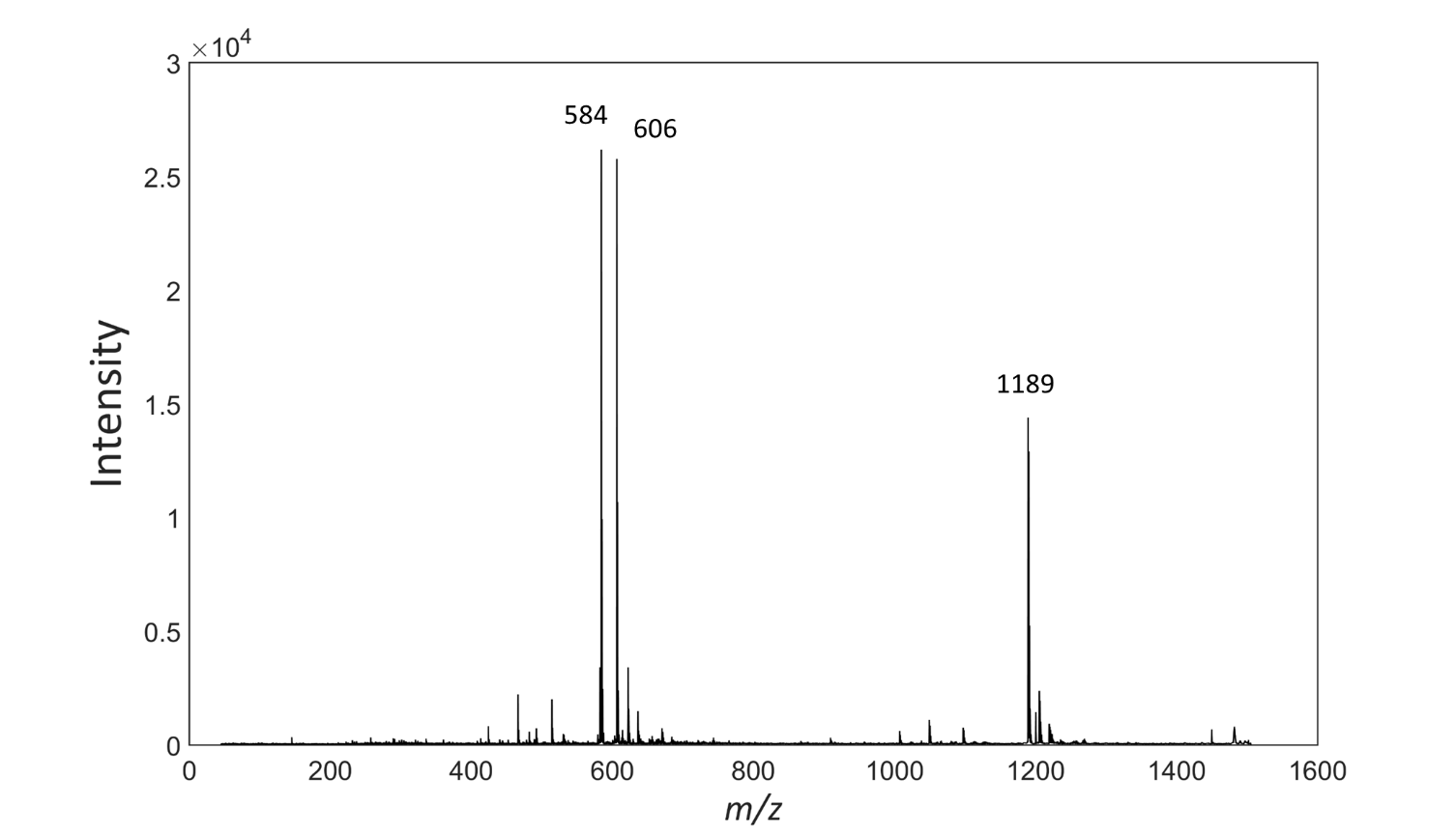


Frag = 90V

**Figure S4b.** ESI(-)MS spectrum of denigrin E (**4**)


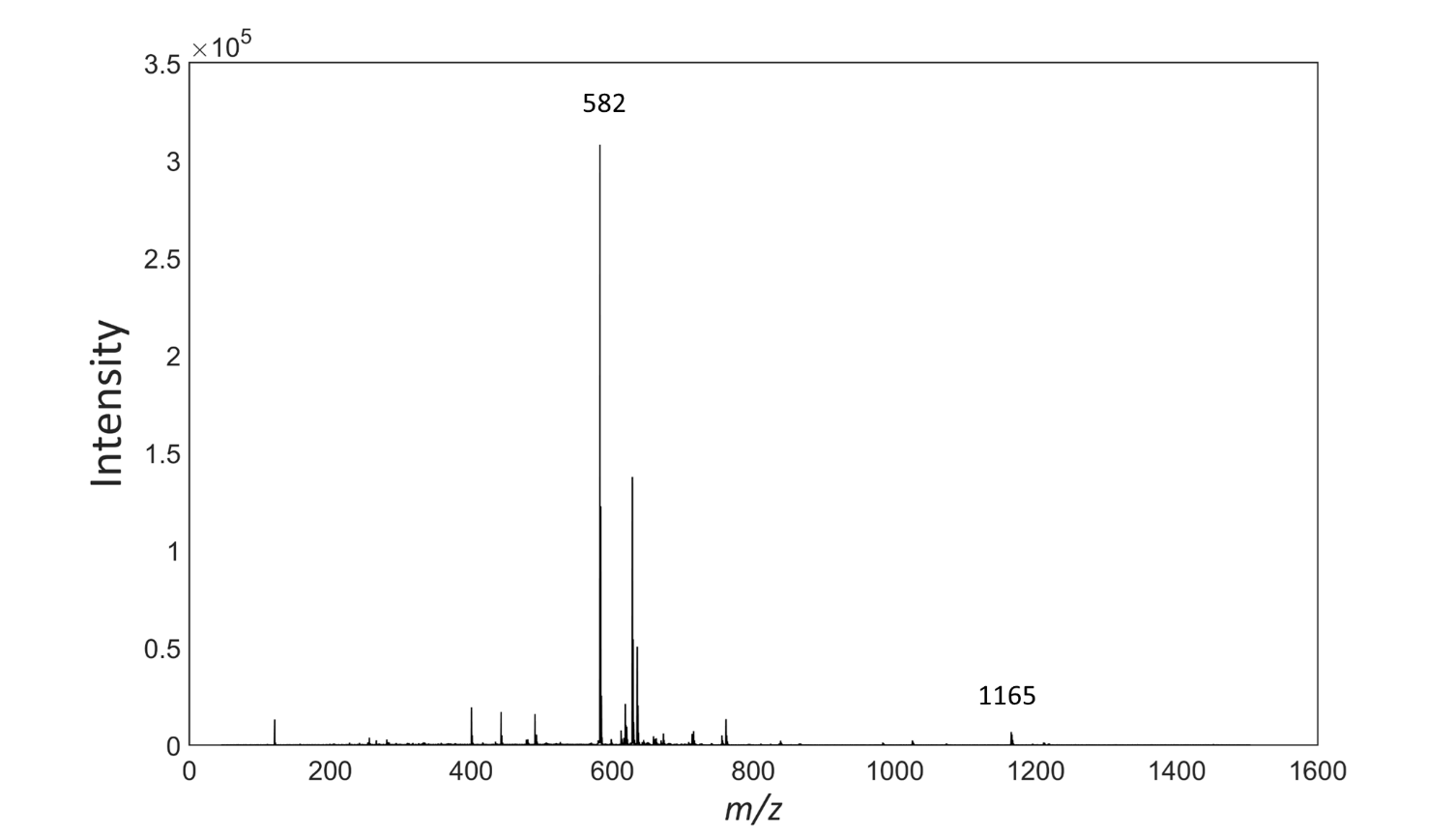


Frag = -150V

**Figure S5a.** ESI(+)MS spectrum of dactypyrrole A (**5**)


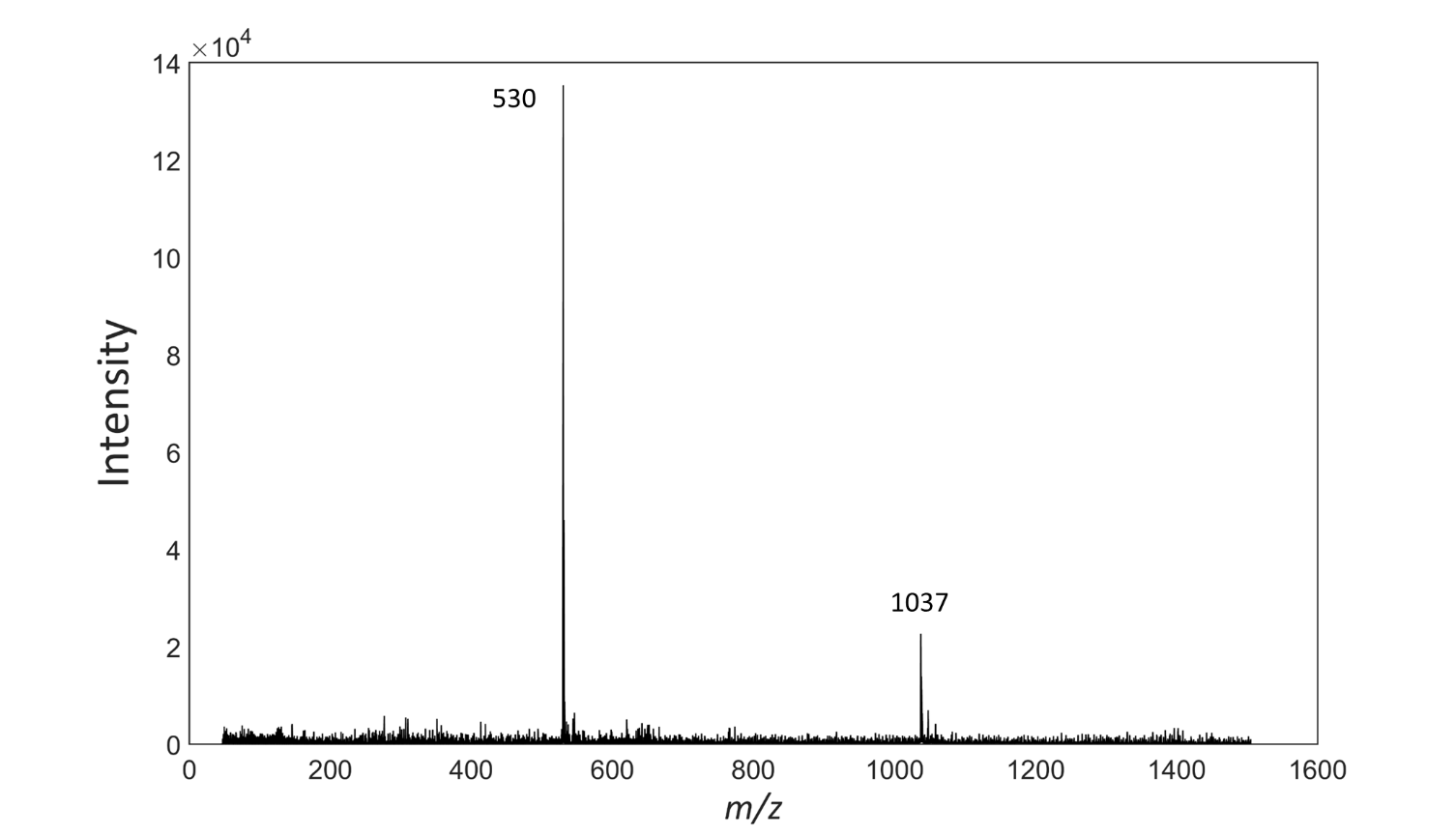


Frag = 150V

**Figure S5b.** ESI(-)MS spectrum of dactypyrrole A (**5**)


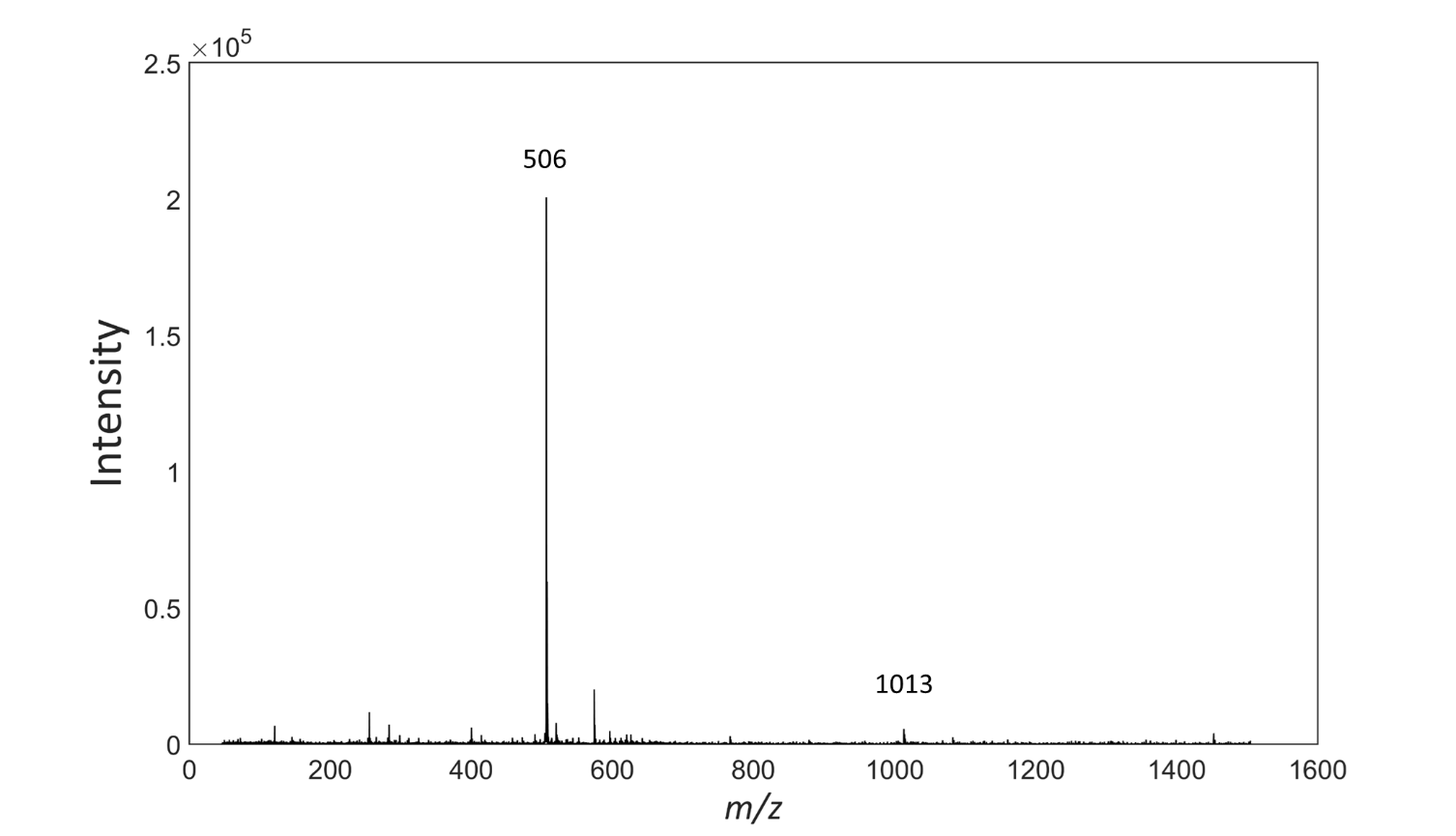


Frag = -150V

**Figure S6a.** ESI(+)MS spectrum of lamellarin O1 (**6**)


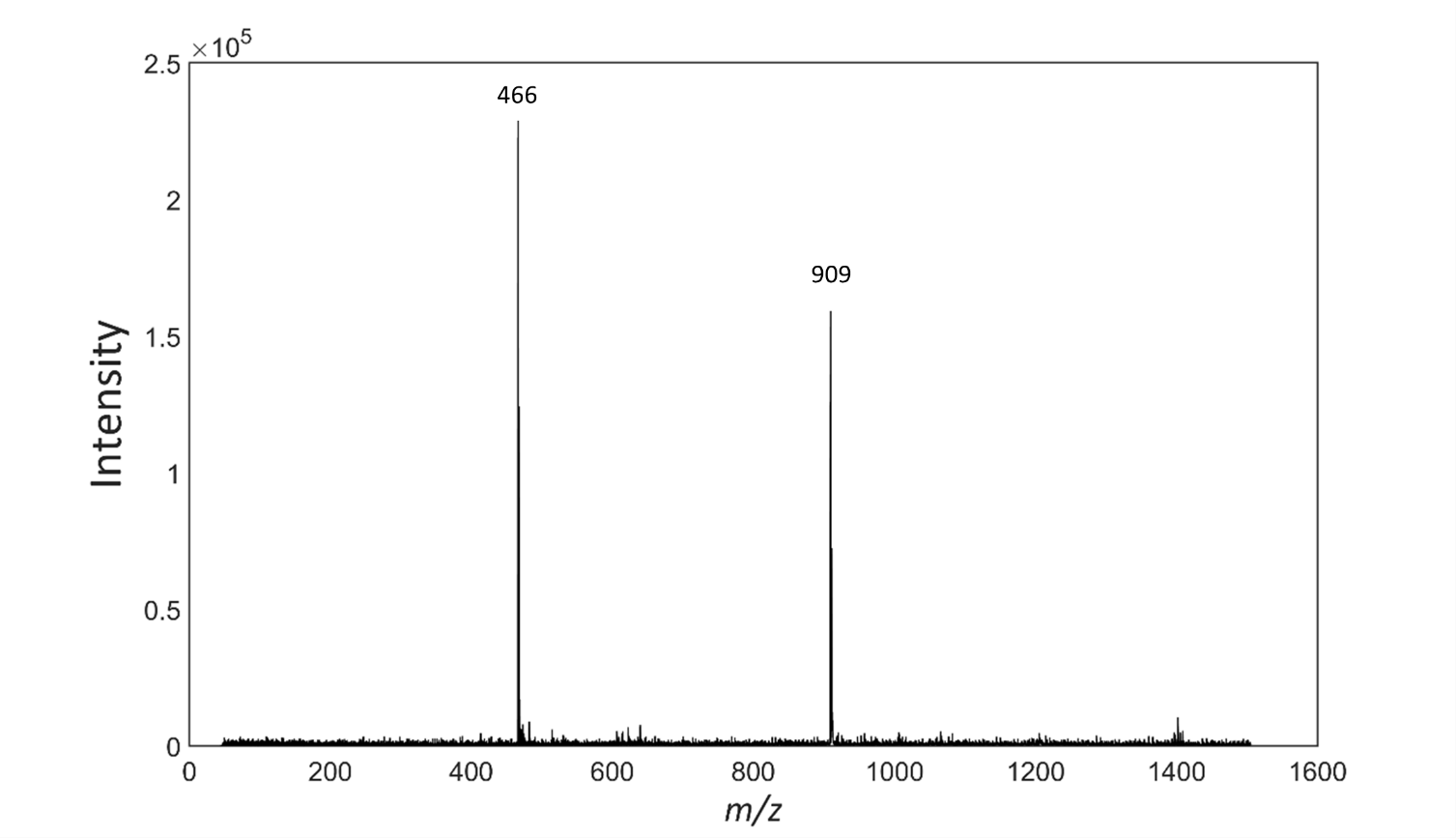


Frag = 150V

**Figure S6b.** ESI(-)MS spectrum of lamellarin O1 (**6**)


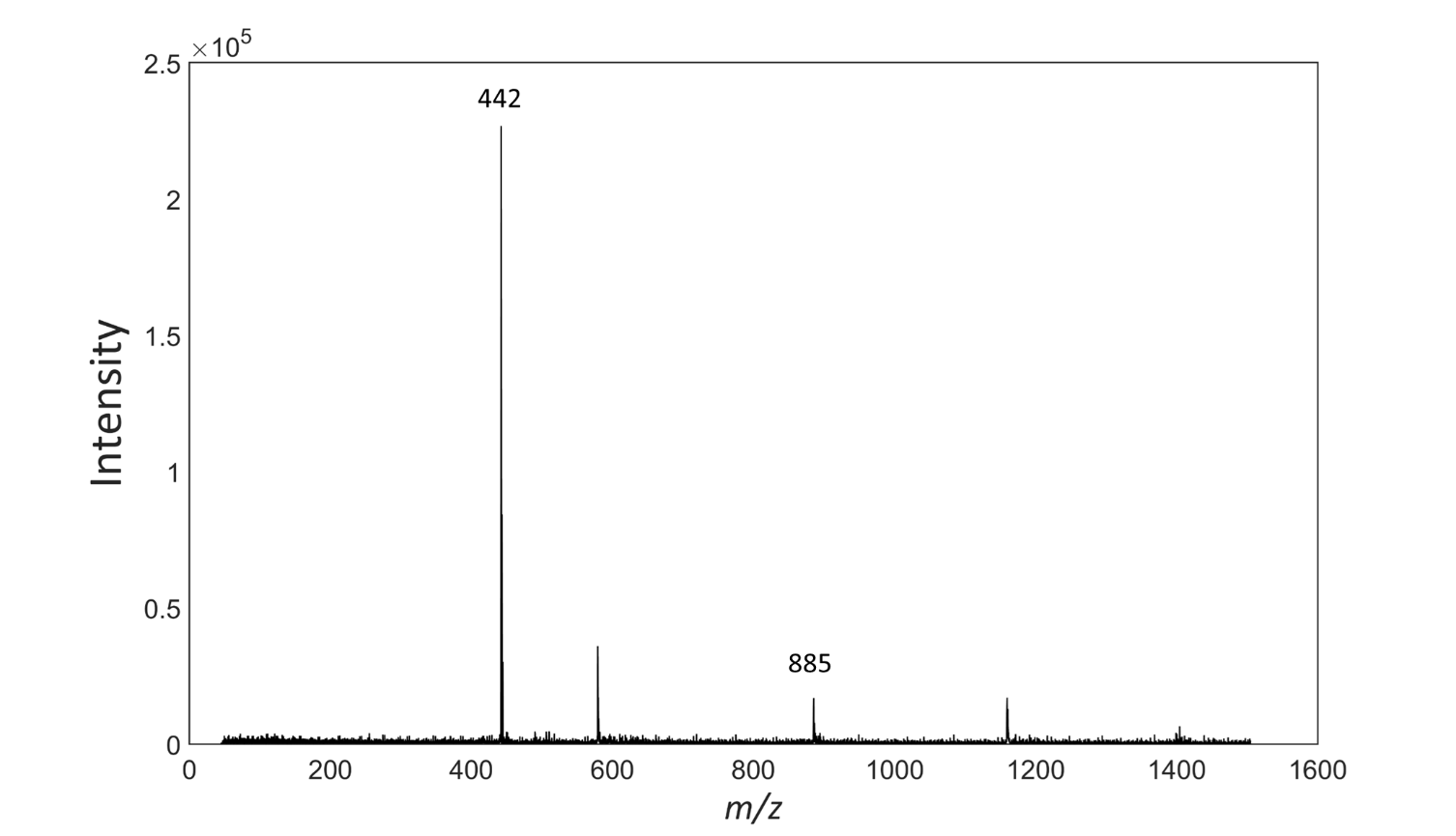


Frag = -150V

**Figure S7.** ESI(+)MS spectrum of 5α,8α-epidioxy-24-methylcholesta-6, 22-dien-3β-ol (**7**)


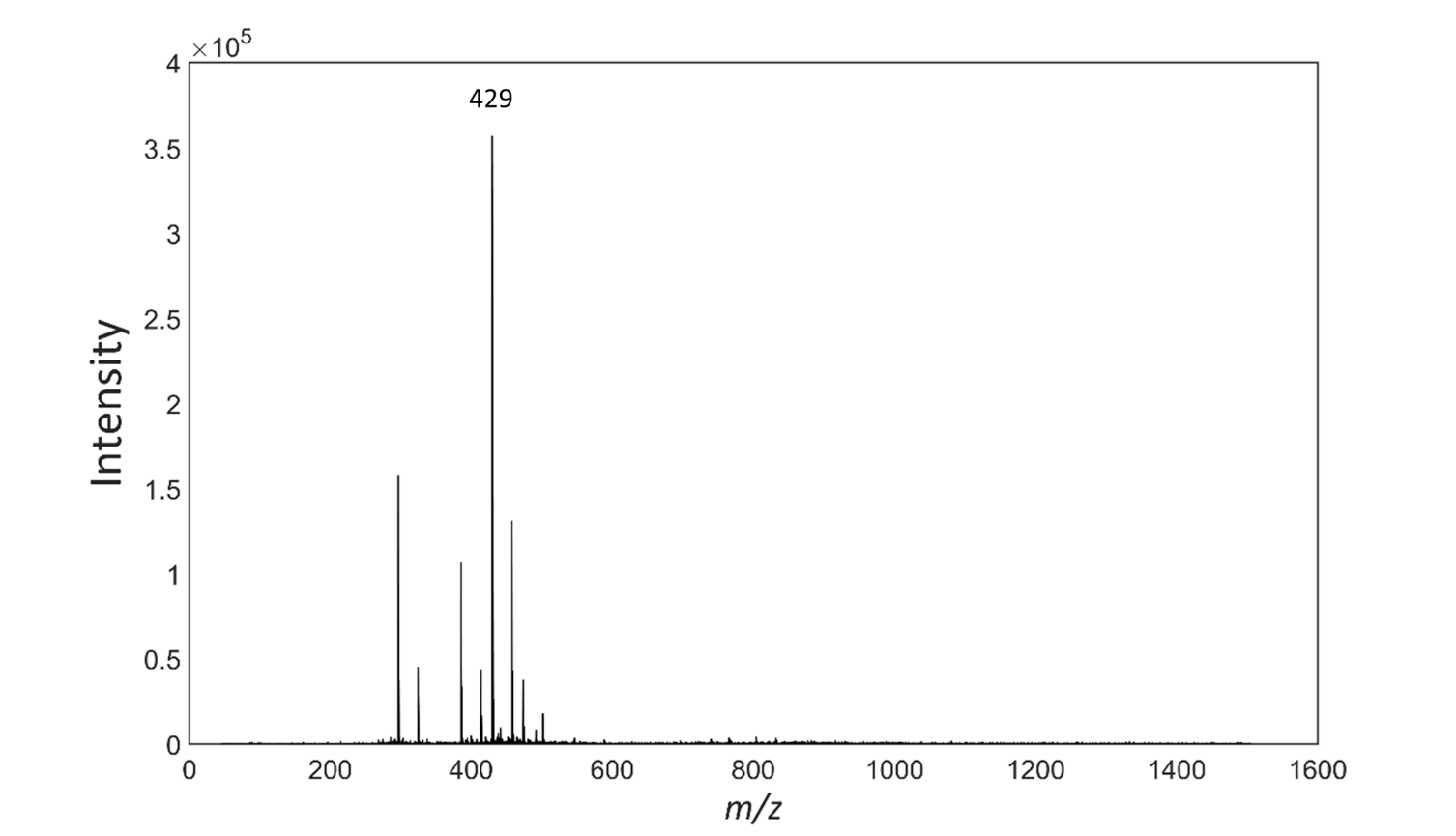


Frag = 120V
